# Allosteric mutants show that PrfA activation is dispensable for vacuole escape but required for efficient spread and *Listeria* survival *in vivo*

**DOI:** 10.1111/j.1365-2958.2012.08121.x

**Published:** 2012-08

**Authors:** Caroline Deshayes, Magdalena K Bielecka, Robert J Cain, Mariela Scortti, Aitor de las Heras, Zbigniew Pietras, Ben F Luisi, Ricardo Núñez Miguel, José A Vázquez-Boland

**Affiliations:** 1 Microbial Pathogenesis Unit, Centres for Infectious Diseases and Immunity, Infection & Evolution, University of EdinburghEdinburgh, UK; 2 Departamento de Bioquímica y Biología Molecular IV, Universidad ComplutenseMadrid, Spain; 3 Department of Biochemistry, University of CambridgeTennis Court Road, Cambridge, UK; 4 Grupo de Patogenómica Bacteriana, Universidad de LeónLeón, Spain

## Abstract

The transcriptional regulator PrfA controls key virulence determinants of the facultative intracellular pathogen *Listeria monocytogenes*. PrfA-dependent gene expression is strongly induced within host cells. While the basis of this activation is unknown, the structural homology of PrfA with the cAMP receptor protein (Crp) and the finding of constitutively activated PrfA* mutants suggests it may involve ligand-induced allostery. Here, we report the identification of a solvent-accessible cavity within the PrfA N-terminal domain that may accommodate an activating ligand. The pocket occupies a similar position to the cAMP binding site in Crp but lacks the cyclic nucleotide-anchoring motif and has its entrance on the opposite side of the β-barrel. Site-directed mutations in this pocket impaired intracellular PrfA-dependent gene activation without causing extensive structural/functional alterations to PrfA. Two substitutions, L48F and Y63W, almost completely abolished intracellular virulence gene induction and thus displayed the expected phenotype for allosteric activation-deficient PrfA mutations. Neither PrfA^allo^ substitution affected vacuole escape and initial intracellular growth of *L. monocytogenes* in epithelial cells and macrophages but caused defective cell-to-cell spread and strong attenuation in mice. Our data support the hypothesis that PrfA is allosterically activated during intracellular infection and identify the probable binding site for the effector ligand. They also indicate that PrfA allosteric activation is not required for early intracellular survival but is essential for full *Listeria* virulence and colonization of host tissues.

## Introduction

Virulence of the food-borne pathogen *Listeria monocytogenes* depends on its ability to proliferate intracellularly. This is mediated by nine bacterial genes encoding products that promote host cell invasion (internalins InlA and InlB), escape from the phagocytic vacuole [pore-forming toxin listeriolysin O (LLO), phospholipases C PlcA and PlcB, protease Mpl], rapid replication in the cytosol (sugar phosphate permease Hpt) and direct cell-to-cell spread (actin-polymerizing surface protein ActA, small internalin InlC) (Vazquez-Boland *et al*., 2001; Portnoy *et al*., 2002; Hamon *et al*., 2006; Cossart, 2011). These virulence genes form a regulon under the positive control of the PrfA protein (Scortti *et al*., 2007). *prfA* null mutants are avirulent (Mengaud *et al*., 1991; Chakraborty *et al*., 1992), reflecting the key role of the PrfA regulator in *Listeria* pathogenesis.

PrfA is a member of the cAMP receptor protein (Crp)/fumarate nitrate reductase regulator (Fnr) family of bacterial transcription factors. Its three-dimensional structure is similar to that of *Escherichia coli* Crp (aka catabolite activator protein, CAP) (Eiting *et al*., 2005). Both PrfA and Crp are homodimers with protomers organized in two domains: N-terminal, with an eight-stranded antiparallel jelly-roll β-barrel, which in Crp accommodates the binding pocket for its allosteric activator, cAMP; and C-terminal, in which the DNA-binding helix–turn–helix (HTH) motif is located. These two domains are connected by a long α-helix (αC) that abuts the β-barrel and provides most of the dimer interface. PrfA differs from Crp in that it possesses a unique 25-residue C-terminal extension comprising three short α-helices (αGHI) wedged between the N- and C-terminal domains. This feature is thought to stabilize the protomer (Vega *et al*., 2004; Eiting *et al*., 2005), possibly explaining why native PrfA exhibits detectable (albeit weak) sequence-specific DNA binding activity (Vega *et al*., 1998; Bockmann *et al*., 2000; Eiting *et al*., 2005), in contrast to apo-Crp, which is totally inactive (Harman, 2001). The symmetrical PrfA dimer activates transcription by binding through its HTH pair to a palindromic ‘PrfA box’ with consensus sequence tTAACanntGTtAa, centred on position −41.5 in the target promoters (Scortti *et al*., 2007).

*Listeria monocytogenes*, a soil-dwelling organism, selectively activates its virulence genes during the transition from environmental saprotroph to intracellular parasite (Freitag *et al*., 2009; de las Heras *et al*., 2011). Sensing a warm-blooded host acts as a primary cue (Leimeister-Wachter *et al*., 1992) via an RNA thermoswitch that prevents translation of the *prfA* transcript at temperatures ≤ 30°C (Johansson *et al*., 2002). However, this mechanism alone is insufficient since the PrfA regulon is only weakly expressed *in vitro* in rich culture media at 37°C (Ripio *et al*., 1996). High levels of expression are observed during intracellular infection (Moors *et al*., 1999; Renzoni *et al*., 1999; Shetron-Rama *et al*., 2002; Chatterjee *et al*., 2006; Joseph *et al*., 2006), suggesting that a host cell-derived signal(s) is required for full PrfA regulon activation.

It has been hypothesized that PrfA, like other Crp/Fnr family members, is allosterically regulated (Vega *et al*., 1998; 2004; Eiting *et al*., 2005) and that this may play a key role in the intracellular activation of PrfA-dependent virulence genes (Scortti *et al*., 2007; de las Heras *et al*., 2011). Evidence that PrfA can alternate between two states, weakly active (OFF), as the native wild-type protein, and strongly active (ON), was provided by the identification of PrfA* substitutions that increase the specific DNA-binding activity (Ripio *et al*., 1997; Shetron-Rama *et al*., 2003; Vega *et al*., 2004; Miner *et al*., 2008). *L. monocytogenes* bacteria carrying a prfA* allele (e.g. prfA*^G145S^) constitutively overexpress all PrfA-dependent genes *in vitro*. The levels of expression are similar to those of intracellular wild-type *L. monocytogenes* (de las Heras *et al*., 2011 and our data herein), suggesting that PrfA shifts during infection to an ‘ON state’ similar to that of the PrfA*^G145S^ protein.

In this study, we examined the possible involvement of the PrfA N-terminal domain in allosteric signalling based on its structural similarities with the Crp cAMP-binding domain. By computational prediction of functional sites and solvent-accessible routes in the protein structure, we identified and mapped an internal pocket within the PrfA N-terminal domain β-barrel. We assessed the capacity of this pocket to influence PrfA-dependent gene activation within host cells by site-directed mutagenesis. Our findings suggest that the strong induction of virulence genes seen in intracellular *L. monocytogenes* involves the allosterically regulated switching of PrfA to a highly active conformation, presumably via binding of an effector molecule in the identified pocket. The targeted pocket mutants allowed us to analyse the role of PrfA allostery in *L. monocytogenes* virulence. We show that PrfA activation is not required for the early events of listerial intracellular infection (phagosome escape and initial replication) but essential for efficient cell-to-cell spreading and *in vivo* survival.

## Results

### Identification of an accessible pocket in the N-terminal domain of PrfA

**Fig. 1:**
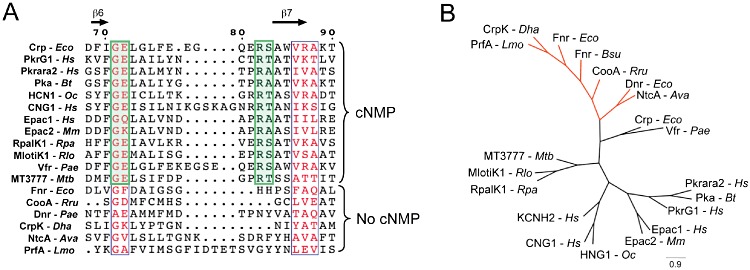
The ‘phosphate binding cassette’ (PBC) is absent from PrfA and other CNBD-containing proteins not regulated by cyclic nucleotide monophosphates (cNMP). A. Amino acid sequence alignment of the CNBD β6–β7 region in a selection of bacterial and eukaryotic proteins regulated and not regulated by cNMP. Conserved blocks of sequence are shown in red and residues known to interact with the cNMP molecule are shaded green. Numbering above the sequences corresponds to Crp, the position of β-strands 6 and 7 of the β-barrel is indicated by arrows. clustalw2 alignment (http://www.ebi.ac.uk/Tools/clustalw2/) visualized with ESPript (http://espript.ibcp.fr/; Gouet *et al*., 1999). cNMP-regulated proteins: *E. coli* Crp/Cap (P0ACK0); *Homo sapiens* PrkG1 (Q13976), Prkar2A (P13861, CNG1 (P29973) and Epac1 (O95398); *Bos taurus* Pka (P00514); *Oryctolagus cuniculus* HCN1 (Q9MZS1); *Mus musculus* Epac2 (Q9EQZ6); *Rhodopseudomonas palustris* RpalK1 (Q02006); *Rhizobium loti* MlotiK1 (Q98GN8); *Pseudomonas aeruginosa* Vfr (P55222); *Mycobacterium tuberculosis* MT3777 (O69644). cNMP-non-regulated proteins and corresponding effector ligands: *E. coli* Fnr (P0A9E5), oxygen (Green *et al*., 2001); *Rhodospirillum rubrum* CooA (P72322), haem-CO (Lanzilotta *et al*., 2000); *P. aeruginosa* Dnr (Q51441), haem-NO (Giardina *et al*., 2008); *Desulfitobacterium hafniense* CprK (B8FW11), *ortho*-chlorophenolacetic acid (Levy *et al*., 2008); *Anabaena variabilis* NtcA (P0A4U7), 2-oxoglutarate (Vazquez-Bermudez *et al*., 2002); *L. monocytogenes* PrfA (P22262), unknown. EMBL accession numbers in parentheses. B. Unrooted neighbour-joining phylogenetic tree of sequences included in the alignment shown in (A). The branch containing only cNMP-non-regulated CNBD proteins is shown in red. Constructed with Phylip 3.69 (http://evolution.genetics.washington.edu/phylip.html) and visualized using FigTree (http://tree.bio.ed.ac.uk/software/figtree/).

The jelly-roll β-barrel fold that forms most of the PrfA N-terminal domain is a key structural component of the cyclic nucleotide monophosphate (cNMP)-binding domain (CNBD). The CNBD is a conserved ≍ 120-residue signalling element present in evolutionarily and functionally diverse proteins including eukaryotic regulators (protein kinases A and G, guanine nucleotide exchange factor Epac), eukaryotic and prokaryotic ion channels, and bacterial transcription factors (Fig. S1). CNBD modules are not only found in cAMP/cGMP-regulated proteins but also in proteins regulated by other ligands (Berman *et al*., 2005; Kannan *et al*., 2007; Rehmann *et al*., 2007; Kornev *et al*., 2008). CNBDs from cNMP-regulated proteins possess the phosphate binding cassette (PBC) (Figs 1A and S1), a signature sequence located between β-strands 6 and 7 of the β-barrel with key residues for phosphoribose docking (Diller *et al*., 2001; Berman *et al*., 2005; Kannan *et al*., 2007; Rehmann *et al*., 2007). A phylogenetic tree analysis of the region flanked by CNBD β6 and β7 from proteins regulated by cNMPs or by other effector molecules showed that the PrfA sequence clusters together with the latter in a group characterized by the absence of a conserved PBC (Fig. 1B). Thus, while cNMPs do not appear to be the PrfA-activating ligands, as indicated by earlier experimental observations (Vega *et al*., 1998), the features of the PrfA N-terminal domain suggest it may bind an unidentified modulator.

**Fig. 2:**
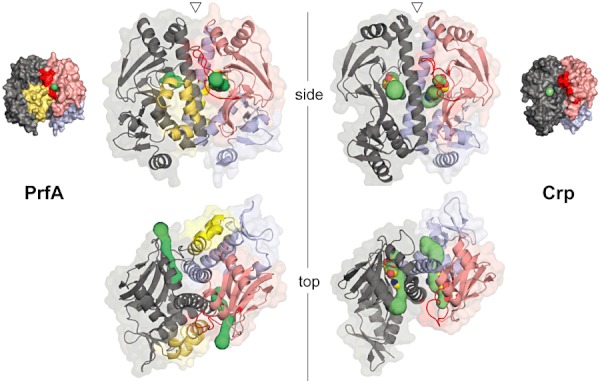
Position and trajectories of the PrfA N-terminal domain pocket and Crp cAMP-binding pocket. Ribbon representation (with surface in transparency) of ‘side’ and ‘top’ views of the crystal structures of PrfA (PDB code 2BEO) and the cAMP–Crp complex (PDB code 1G6N). The solvent-accessible access channel as determined by caver (Petrek *et al*., 2006) is shown in green. A smaller ‘side’ view of the PrfA and Crp dimers in surface-only representation is shown for reference. The ‘top’ view is a 90° rotation with the arrowhead brought to the foreground. In both structures, one protomer is coloured light grey with the N-terminal domain in pink, the other protomer is dark grey. The PBC loop (and corresponding region in PrfA) is in red. The additional C-terminal GHI helical bundle of PrfA is in yellow. In Crp, the cAMP molecule is in sphere representation with atoms coloured by element (C, yellow; O, red; N, blue).

**Fig. 3:**
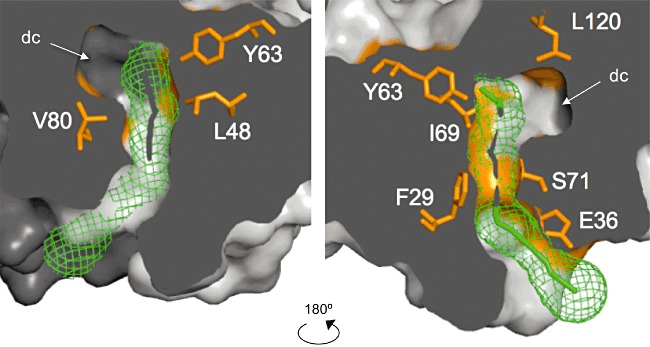
Site-directed mutagenesis of surface-exposed residues of the PrfA N-terminal domain pocket. Mutated residues are represented as orange sticks in a detail of the cutaway cross-section of the PrfA dimer shown in Fig. S4A, in which the two halves of the internal surface of one of the pockets is shown. The green volume in mesh representation is the pocket trajectory determined by caver (Petrek *et al*., 2006). Note that the caver graphical output represents the solvent access path and does not fill the entire volume of the distal chamber's (dc) cavity. See also Fig. S5 for additional reference.

Superimposition of the structures of PrfA and the cAMP–Crp complex revealed a sizable void within the PrfA β-barrel at about the same position as the Crp cAMP-binding pocket (Fig. S2). Residues lining this internal cavity were independently identified using the Functional Protein Sequence Pattern (fpsp) program (Núñez Miguel, 2004) to predict solvent-accessible regions in PrfA that are likely to form functionally important interactions (patterns 62-YYKGAFVI-69 and 69-IMSGFIDTETSVGYY-83). The pathway leading from this interior pocket to the outside bulk solvent was mapped with the program caver (Petrek *et al*., 2006) (Fig. 2). The PrfA pocket forms an elongated, irregular channel with an average inner width of ≍ 4.5 Å that extends from the protein surface deep into the β-barrel. The distal portion opens onto a wider chamber of ≍ 4.5 Å × 7 Å × 10 Å that ends between the two αC helices of the PrfA dimer (αC and αC′ from the opposite monomer), halfway along their length where the helical axes cross (Figs 2, 3 and S3A). Both αC and αC′ contribute residues to form the walls of the distal chamber. The topology of the pocket does not change significantly between the crystal structures of PrfA^WT^ and the constitutively activated PrfA*^G145S^ mutant (Fig. S3). The pocket surface is mostly non-polar except at the mouth and proximal section of the channel tract, where there is an electronegative patch, and in portions of the distal chamber where the polar substituents of the side-chains of Y63, Q121 and Q123 line the cavity (Fig. S4).

While similarly located, the PrfA pocket and the Crp cAMP binding site differ in shape and trajectory. The latter traverses the β-barrel from side to side, with a small second opening at the bottom close to the PBC where the cAMP polar head lodges, whereas the PrfA pocket is shorter and ends at the monomer–monomer interface. Moreover, the mouth of the PrfA pocket opens out on the opposite side of the β-barrel, between the loop that forms the PBC in Crp and an adjacent loop between β-strands 2 and 3. The region that corresponds to the pocket entrance in Crp is occluded in PrfA by the additional C-terminal GHI α-helical bundle that is wedged between the two protomer domains (Fig. 2).

### Site-directed mutagenesis of the PrfA pocket

If the identified pocket accommodates a ligand that activates or stabilizes PrfA in highly active (ON) conformation, amino acid substitutions within it are expected to impair the intracellular induction of PrfA-dependent genes. We selected residues lining the cavity with solvent-exposed side-chains and introduced substitutions that alter the pocket with minimal secondary structure consequences. Seven substitutions were chosen to change the shape of the pocket, without significantly altering the local hydrophobicity, in the proximal (F29M), middle (S71L) and distal portions (L48F, Y63W, I69W and V80L) of the channel and end of the inner chamber (L120V). Two of them (S71L and Y63W) also removed hydroxyl groups that could make polar contacts with a potential ligand. Two other substitutions targeted the negatively charged patch at the pocket entrance (E36Q, E36R) (Figs 3, S4 and S5).

The *prfA* alleles were stably inserted in monocopy in the chromosome of a *L. monocytogenes*Δ*prfA* strain (see *Experimental procedures*). *prfA* can be expressed from its own two constitutive promoters (P1*prfA* and P2*prfA*) or the PrfA-regulated promoter of the upstream *plcA* gene (P*plcA*). P*plcA* thus creates a positive autoregulatory loop (Mengaud *et al*., 1991), although negative feedback has also been reported (Freitag *et al*., 1993; Greene and Freitag, 2003). To isolate and measure accurately the effects of changes in PrfA activity on virulence gene expression without any interference from the P*plcA-*driven PrfA autoregulatory loop, we expressed *prfA* from P1/P2*prfA*. Immunoblotting of bacterial whole-cell extracts verified that all complemented Δ*prfA* bacteria produced similar amounts of PrfA protein (not shown). As controls, Δ*prfA* was complemented with an empty vector, the constitutively hyperactive prfA*^G145S^ allele and its DNA binding-deficient prfA*^sup^ derivative (PrfA* suppressor substitution E173G in the HTH motif) (Vega *et al*., 2004). The complemented Δ*prfA* strains (hereafter designated by their *prfA* allele) were grown in brain heart infusion (BHI) broth, in which PrfA-dependent genes are weakly expressed (basal levels) (Ripio *et al*., 1997; Vega *et al*., 2004), and in infected HeLa cells, in which PrfA is expected to be predominantly in the highly active (ON) state (de las Heras *et al*., 2011). PrfA activity was determined by measuring expression from the strictly PrfA-dependent promoters P*plcA* and P*actA* (*actA-plcB* operon) (Lalic-Multhaler *et al*., 2001; Vega *et al*., 2004) by reverse-transcription quantitative real-time PCR (RT-QPCR).

**Fig. 4:**
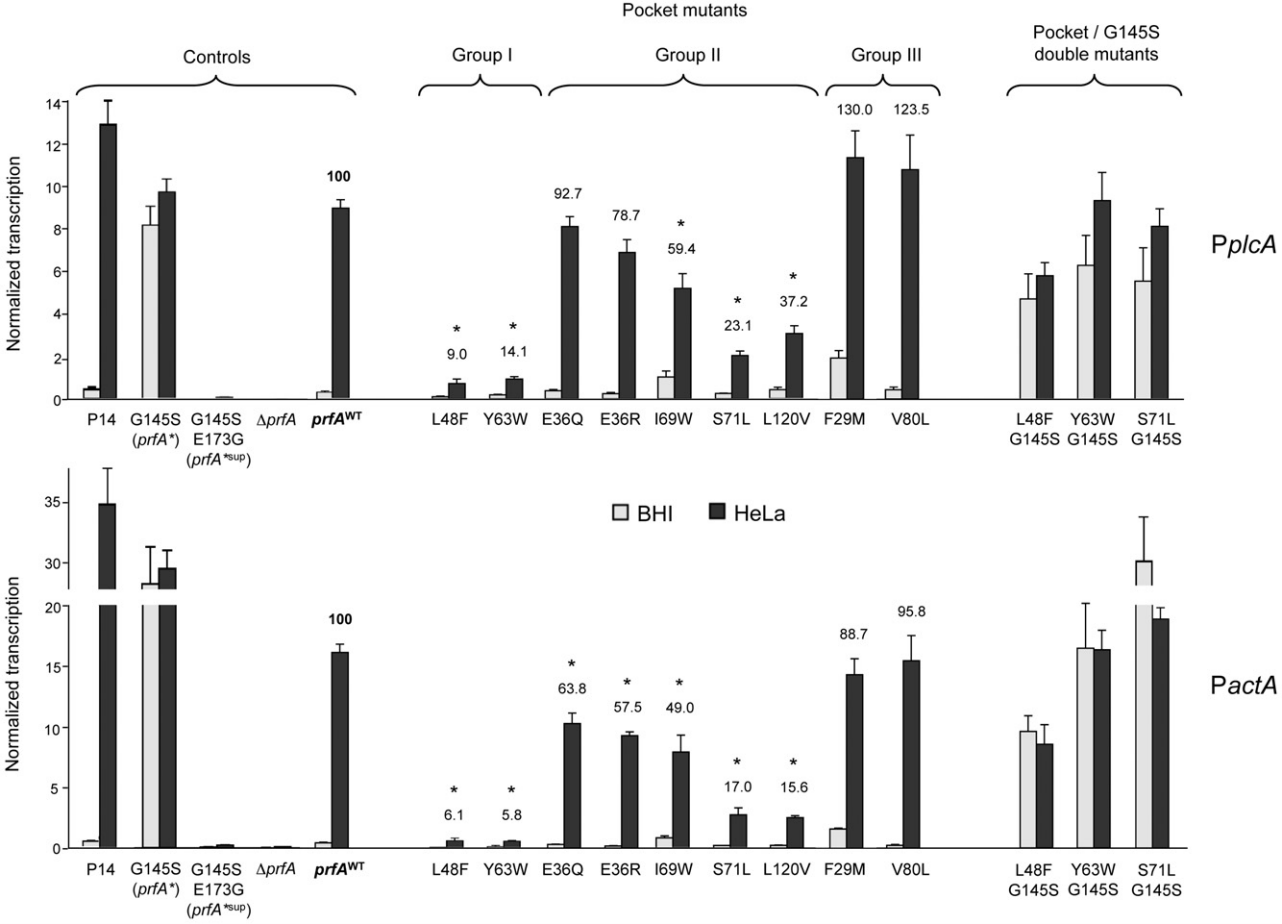
Effect of PrfA pocket substitutions on intracellular virulence gene activation. Expression from the PrfA-regulated promoters P*plcA* and P*actA* was analysed by RT-QPCR in extracellular and intracellular conditions [respectively: exponentially growing BHI culture (OD_600_ = 0.3) and HeLa cells infected for 6 h]. *L. monocytogenes*Δ*prfA* was complemented with the following *prfA* constructs: Δ*prfA*, vector with no insert; *prfA*^WT^, wild-type allele; substitutions in single-letter code, *prfA* pocket mutant alleles; see Table S2. P14 is the isogenic wild-type parent strain of the Δ*prfA* strain used to express the *prfA* constructs. Numbers above bars, per cent intracellular activation relative to *prfA*^WT^. Statistically significant differences (*P* ≤ 0.01) indicated by asterisks. Mean of at least three independent experiments ± SEM.

PrfA-dependent expression was strongly activated within host cells in *prfA*^WT^ (14.5- and 34.1-fold compared with BHI levels for P*plcA* and P*actA* respectively). Albeit lower, this induction was comparable to that observed for the isogenic wild-type parent strain with a native, autoregulated *prfA* locus (strain P14). No PrfA-dependent gene expression was detected in the negative controls either extracellularly (BHI) or intracellularly (Fig. 4).

The pocket mutants could be grouped into three categories according to strength of intracellular PrfA-dependent gene activation (Fig. 4). Group I mutants (*prfA*^L48F^ and *prfA*^Y63W^) showed virtually abolished PrfA inducibility and were therefore defined as activation-deficient (mean reduction relative to *prfA*^WT^: P*plcA* 86%, P*actA* 94%). Group II mutants displayed different degrees of impaired PrfA-dependent gene activation: strong (*prfA*^S71L^ and *prfA*^L120V^; mean reduction: P*plcA* 70%, P*actA* 84%), intermediate (*prfA*^I69W^; P*plcA* 41%, P*actA* 51%) and modest (*prfA*^E36Q/R^; significantly reduced inducibility only for P*actA*, 36% and 43% respectively). Group III mutants (*prfA*^F29M^ and *prfA*^V80L^) behaved similarly to *prfA*^WT^.

The prfA*^G145S^ strain exhibited high expression levels in both conditions that were as great as those for *prfA*^WT^ intracellularly (Fig. 4). This is consistent with PrfA adopting an ON conformation within host cells functionally similar to that of the constitutively hyperactive PrfA*^G145S^ mutant protein.

*prfA*^F29M^ and *prfA*^I69W^ showed comparatively elevated levels of basal (BHI) PrfA-dependent expression (relative to *prfA*^WT^: P*plcA* 270% and 140%, P*actA* 312% and 145% respectively; Fig. 4) and displayed a weak PrfA* phenotype on egg yolk agar (Fig. S6). A third mutant, *prfA*^S71L^, also exhibited weak PrfA* phenotype on agar plates (Fig. S6) but it did not differ in basal expression with *prfA*^WT^ by RT-QPCR (Fig. 4). These data suggest that the F29M, I69W and, possibly also, S71L substitutions cause some increase in the intrinsic activity of PrfA.

### Activation deficiency of pocket mutants is not due to structural disruption of PrfA

The activation defects could be due to changes in the pocket interfering with PrfA allostery/OFF-ON equilibrium, or to more extensive structural perturbations in PrfA leading to total loss of function. We followed two approaches to distinguish between these two possibilities.

First, recombinantly expressed purified His-tagged PrfA proteins were subjected to biophysical characterization (Table 1). The oligomeric state of PrfA, essential for function, was assessed by analytical ultracentrifugation (Lebowitz *et al*., 2002). The calculated molecular mass for all the proteins was in agreement with the theoretical molecular weight of the PrfA dimer (54.56 kDa), indicating that none of the pocket substitutions did affect significantly interprotomer association (Table 1). The effect of the mutations on overall PrfA stability was tested using a fluorescence-based thermal shift assay (Ericsson *et al*., 2006). Most pocket substitutions did not significantly alter PrfA melting temperature (*T*_m_). I69W and S71L exhibited a minor reduction in the thermal stability but the values remained within the range of a functional PrfA protein (on the basis of the slightly lower *T*_m_ of PrfA*^G145S^ compared with PrfA^WT^, ≍ 55°C versus ≍ 62°C respectively). A larger decrease was observed for L48F (10.4°C below PrfA^WT^), but the *T*_m_ still approximated that of PrfA*^G145S^ (Table 1). Finally, the specific DNA-binding activity was determined by surface plasmon resonance using 40-bp double-stranded DNA fragments containing the PrfA boxes of P*plcA* (shared by the divergently transcribed PrfA-regulated *hly* gene) and P*actA* (Table 1). PrfA is expected to retain detectable intrinsic (native OFF state) activity if the substitutions do not introduce gross structural distortions in the protein. The calculated equilibrium dissociation constant (*K*_D_) of PrfA^WT^ for the P*plcA*/*hly* box (10.96 ± 0.49 × 10^–7^ M) was very similar to that previously determined by Eiting *et al*. (2005) using the same technique (9 ± 1 × 10^–7^ M). Of all the pocket substitutions, only the activation-deficient L48F associated with a significant reduction in the affinity for both DNA targets, although it retained detectable binding activity for the P*plcA*/*hly* box. The other activation-deficient mutation, Y63W, had no major effect on PrfA intrinsic activity. PrfA^F29M^, PrfA^I69W^ and PrfA^S71L^ had higher than wild-type intrinsic DNA-binding activity for both target sequences (fold difference 3.8–5.1, 3.2–3.5 and 1.6–1.8 respectively), consistent with the partial PrfA* phenotype exhibited by the corresponding bacteria (see above).

**Table 1:** Biophysical characterization of PrfA pocket mutant proteins

					Specific DNA-binding affinity			
					
		Dimerization^c^		Thermal stability	P*plcA*/*hly* box^e^		P*actA* box	
					
PrfA substitution	Description	MW (kDa)	% peak integration	*T*_m_ (°C)	*K*_D_ (nM)	% PrfA^WT^	*K*_D_ (nM)	% PrfA^WT^
None (PrfA^WT^)	Activable	58.3 ± 9.8	95.5	61.8 ± 1.3	1096 ± 49	100	1363 ± 24	100
G145S (PrfA*)	Constitutively activated	54.4 ± 5.6	83.9	54.5 ± 1.0^d^	≤ 1^f^	–	≤ 1^f^	–
G145S/E173G (PrfA*^sup^)	DNA binding-deficient	53.3 ± 3.4	81.3	52.0 ± 0.5	10518 ± 317	9	32022 ± 528	4
F29M	Group III (−)^a^	49.0 ± 14.9	96.0	59.8 ± 0.3	288 ± 45	381	267 ± 15	510
E36Q	Group II (+)	59.4 ± 12.4	85.3	65.5 ± 0.5	1356 ± 11	81	801 ± 67	170
E36R	Group II (++)	52.0 ± 3.0	86.8	66.5 ± 0.5	1445 ± 13	76	1079 ± 10	126
L48F	Group I (++++)	50.4 ± 4.7	90.9	51.3 ± 0.3	5265 ± 101	21	20842 ± 262	7
Y63W	Group II (++++)	50.0 ± 3.0	84.4	59.3 ± 2.0	1158 ± 86	95	1660 ± 14	82
I69W	Group II (++)	49.0 ± 7.8	92.9	57.5 ± 0.0	347 ± 43	316	392 ± 76	348
S71L	Group II (+++)	50.3 ± 3.0	83.3	54.3 ± 0.3	695 ± 42	158	741 ± 59	184
V80L	Group III (−)	52.0 ± 4.9	92.6	62.0 ± 0.0	1785 ± 47	61	1404 ± 54	97
L120V	Group II (+++)	50.7 ± 3.1	92.3	60.2 ± 0.2	3123 ± 37	35	1586 ± 17	86
L48F/G145S	Double mutant^b^	49.8 ± 7.0	93.3	nd	15 ± 1	7362	239 ± 60	571
Y63W/G145S	Double mutant	51.3 ± 3.1	83.0	nd	9 ± 1	11617	29 ± 1	4754
S71L/G145S	Double mutant	51.3 ± 6.3	72.9	nd	75 ± 1	1465	67 ± 1	2039

a. Intracellular activation phenotype grouping for PrfA pocket single substitutions (see Fig. 4): group I = activation-deficient; group II = activation-impaired; group III = wild-type activation. Rating of activation defect relative to PrfA^WT^: (−) = no activation defect; (+) to (++++) = weak to very strong effect.

b. N-terminal domain pocket/C-terminal domain PrfA* G145S double mutant.

c. A dimerization-deficient PrfA mutant was used as negative control (PrfA*^G145S/A129T^, experimentally determined MW = 32.3 kDa; M. K. Bielecka *et al*., in preparation).

d. Note that the *T*_m_ for PrfA*^G145S^ was 7.2°C lower than that for PrfA^WT^, indicating that the structural changes associated with the G145S substitution make the hyperactive mutant protein slightly less stable.

e. This PrfA box is shared by the promoters of the divergently transcribed PrfA-regulated genes *plcA* (PlcA phospholipase) and *hly* (LLO pore-forming toxin).

f. Precise *K*_D_ values could not be determined for the constitutively hyperactive PrfA*^G145S^ mutant; likely due to the avidity for its target DNA sequence, the dissociation rate could not be reliably determined and quantified with the Biacore instrument. An analogous situation was previously reported by Mauder *et al*. (2006) for PrfA*^G145S^.

MW, molecular weight determined by analytical ultracentrifugation. *T*_m_, thermal denaturation midpoint temperature determined by fluorescence enhancement of the hydrophobic reporter dye Sypro Orange. *K*_D_, equilibrium dissociation constant in nM determined using the PrfA boxes of the P*plcA*/*hly* and P*actA* promoters as target sequence (relative DNA-binding affinity expressed as percentage of PrfA^WT^). nd, not determined. Data are average values ± SEM.

The second strategy involved combining the pocket mutations with a PrfA*^G145S^ mutation. The G145S substitution in αD exerts its PrfA-activating effect locally within the C-terminal domain by directly stabilizing the neighbouring HTH motif (Eiting *et al*., 2005). Consequently, if the pocket mutations cause structural changes mainly confined to the N-terminal domain, these should not substantially interfere with the G145S mutation/inactivate the PrfA*^G145S^ protein (approach schematized in Fig. S7). Double mutants were prepared for L48F, Y63W and S71L. These mutants displayed a strong PrfA* phenotype indistinguishable from that of prfA*^G145S^ on egg yolk agar (Fig. S6) and elevated basal (BHI) PrfA-dependent expression by RT-QPCR (Fig. 4). The corresponding PrfA proteins also showed significantly increased DNA-binding activity compared with PrfA^WT^ (Table 1).

Overall, our data indicate that the pocket substitutions do not cause ‘catastrophic’ disruption of the PrfA fold nor transmit wide-ranging conformational alterations to the C-terminal domain, consistent with their effects mostly involving the N-terminal domain pocket.

### Effect of PrfA pocket substitutions in *L. monocytogenes* virulence

**Fig. 5:**
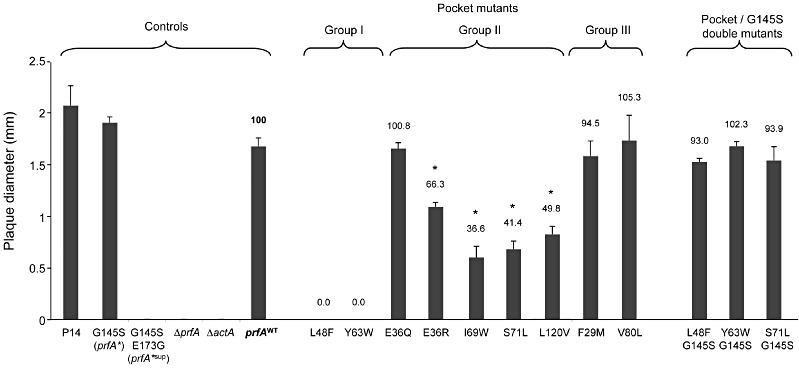
Effect of PrfA pocket substitutions on *L. monocytogenes* cell-to-cell spread. Plaque assay in murine L929 fibroblasts. Bacteria as in Fig. 4. Negative control: isogenic Δ*actA* mutant lacking the actin-polymerizing surface protein ActA essential for cell-to-cell spread (Kocks *et al*., 1992). Numbers above bars, relative spreading of mutants as quantified by average plaque size, expressed in percentage of *prfA*^WT^; see Fig. S8 for representative images of the sizes of the plaques. Mean of at least three independent experiments ± SEM; asterisks, statistically significant differences (*P* ≤ 0.01). Note that despite the marked differences in extracellular (BHI) PrfA-dependent expression levels between *prfA*^WT^ and prfA*^G145S^, both exhibited the same (maximal) levels of spread, supporting the notion that the PrfA*^G145S^ mutant protein mimics the ON (intracellular) PrfA state.

We examined the impact of the pocket mutations on virulence using a plaque assay, which measures the capacity of *L. monocytogenes* to spread in a cell monolayer (Sun *et al*., 1990). Spreading efficiency relies on several different steps of the listerial intracellular infection cycle (phagosome escape, cytosolic replication, actin-based motility/cell-to-cell passage) (Portnoy *et al*., 2002; Hamon *et al*., 2006), all mediated by PrfA-controlled virulence determinants (Scortti *et al*., 2007). The results of the cell-to-cell spread assays were entirely consistent with the intracellular virulence gene expression data: the activation-deficient mutants *prfA*^L48F^ and *prfA*^Y63W^ did not form plaques; the activation-impaired *prfA*^E36R^, *prfA*^I69W^, *prfA*^S71L^ and *prfA*^L120V^ mutants produced smaller plaques concordant with the observed reduction in intracellular PrfA-dependent inducibility; and *prfA*^F29M^, *prfA*^E36Q^ and *prfA*^V80L^, with no or only minor defects in PrfA activation, produced plaques of the same size as *prfA*^WT^ (Fig. 5).

### PrfA L48F and Y63W support normal phagosome escape

**Fig. 6:**
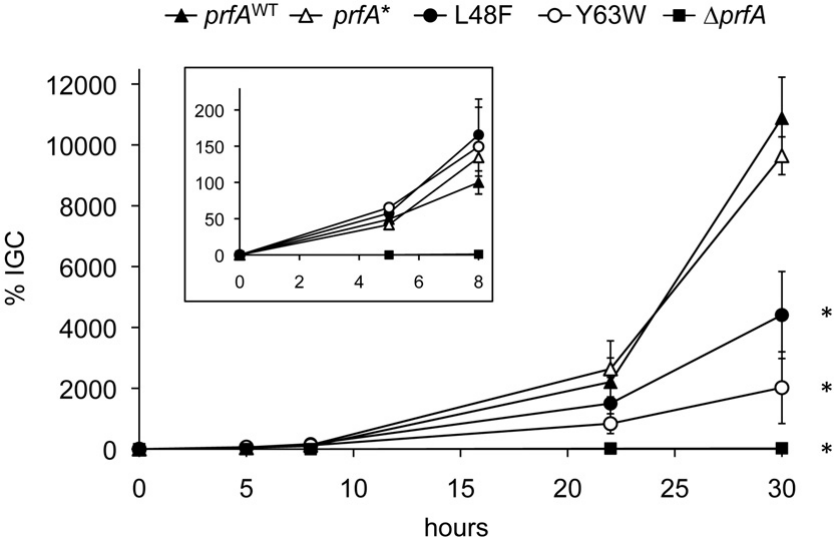
Intracellular growth of *prfA*^allo^ mutants in HeLa cells. *L. monocytogenes*Δ*prfA* complemented with an empty vector or *prfA*^WT^ allele were used as controls. Inset, intracellular growth dynamics at early time points of infection. Data were normalized using an intracellular growth coefficient (IGC) (see *Experimental procedures*) and expressed as per cent of wild type at *t* = 8. Bacterial cfu counts per well at *t* = 0: *prfA*^WT^, 4.3 ± 1.0 × 10^3^; prfA*^G145S^, 13.1 ± 8.4 × 10^3^; *prfA*^L48F^, 2.2 ± 0.3 × 10^3^; *prfA*^Y63W^, 4.4 ± 0.8 × 10^3^; Δ*prfA,* 2.7 ± 0.8 × 10^3^. Mean of at least two duplicate experiments ± SEM. Statistically significant differences (*P* ≤ 0.01) indicated by asterisks.

While directly mediated by the *actA* gene product (Kocks *et al*., 1992), actin-based cell-to-cell spread requires the previous release of bacteria from the phagocytic vacuoles (Portnoy *et al*., 2002). This key step is also promoted by PrfA-dependent virulence determinants (the *hly*-encoded LLO aided by the phospholipases C, particularly PlcB; Vazquez-Boland *et al*., 1992; Marquis *et al*., 1995; Gründling *et al*., 2003) and is essential for intracellular survival and cytosolic replication (Schnupf and Portnoy, 2007). To pinpoint at which phase of the intracellular infection cycle PrfA activation is important, we compared the intracellular proliferation phenotype in HeLa cells of the activation-deficient *prfA*^L48F^ and *prfA*^Y63W^ mutants to that of *prfA*^WT^ and the constitutively activated prfA*^G145S^ mutant (Fig. 6).

Interestingly, while the control Δ*prfA L. monocytogenes* bacteria were unable to grow intracellularly, both *prfA*^L48F^ and *prfA*^Y63W^ appeared to proliferate normally over a standard infection course of 8 h, suggesting they were not affected in phagosome escape. However, a proliferation deficiency became apparent for *prfA*^L48F^ and *prfA*^Y63W^ at later time points of intracellular infection (30 h) (Fig. 6). These data suggest that full activation of PrfA is not necessary for phagosome escape and early intracellular growth, but is required for sustained long-term proliferation, which obviously depends on the capacity of the bacteria to colonize new cells in the monolayer via cell-to-cell spreading.

**Fig. 7:**
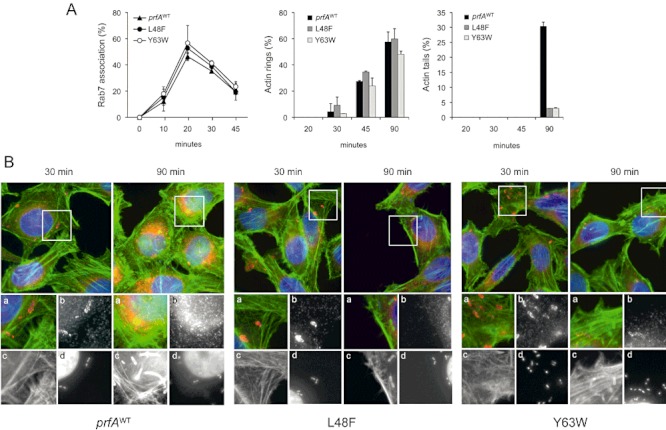
Vacuole escape of *prfA*^allo^ mutants in HeLa cells. A. Vacuole escape dynamics for *prfA*^WT^ and *prfA*^allo^ L48F and Y63W strains determined by fluorescence microscopy. Infected HeLa cells were quantified for association with vacuolar (Rab7) or cytosolic (F-actin rings and tails) markers in an intracellular infection time-course. Mean of three independent experiments ± SEM. B. Representative fluorescence micrographs used to quantify vacuole escape in (A). White boxes in top panels (Rab7, F-actin and DAPI staining merge) indicate areas of interest and are shown as 2.5× magnified sections below: (a) image of boxed area; (b) Rab7 vacuole staining, clearly evident around bacteria at 30 min but not at 90 min; (c) F-actin staining, which is not associated with bacteria at 30 min but present as actin rings at 90 min for all strains, and as actin tails for *prfA*^WT^ but not *prfA*^allo^ (L48F and Y63W) strains; (d) DAPI staining, showing internalized bacteria. Data for each time point are the mean percentage of five microscopic fields per experiment, and three independent experiments. Images were originally captured at 630× magnification.

We directly tested the ability of the *prfA*^L48F^ and *prfA*^Y63W^ alleles to mediate escape from the phagocytic vacuole by quantifying the proportion of vacuolar and cytosolic *Listeria* using fluorescence microscopy over an infection time-course in HeLa cells. The former were identified using the endosomal marker Rab7, present in the *Listeria*-containing vacuole (LCV) just prior to escape (Henry *et al*., 2006). The dynamics of association with Rab7 was essentially identical for *prfA*^WT^, *prfA*^L48F^ and *prfA*^Y63W^, with a peak of 50–60% of bacteria by 20 min after infection and progressive drop to ≍ 20% over the following 25 min (Fig. 7). This decline was not observed at the equivalent time point in a control Δ*hly* mutant (Fig. S9). Cytosolic bacteria were identified by AlexaFluor-conjugated phalloidin to visualize association with F-actin. Two distinct patterns were observed for ‘actin rings’ surrounding the bacteria, indicative of presence in the cytosol, and ‘actin tails’ extending from one bacterial pole, indicative of actin-based motility (Dabiri *et al*., 1990; Mounier *et al*., 1990). Consistent with the Rab7 data, association with F-actin rings did not differ between *prfA*^WT^, *prfA*^L48F^ and *prfA*^Y63W^, with significant staining occurring from 45 min of infection onwards (Fig. 7), as previously reported (Henry *et al*., 2006). Similar results were obtained using a complementary approach for determination of cytosolic bacteria, based on semi-quantitative assessment of decoration with a *Listeria*-specific cytosolic probe expressed from a mammalian expression plasmid (Fig. S10). This probe is the cell wall-binding domain (CBD) from the *L. monocytogenes* phage A118 endolysin, fused to yellow fluorescent protein (YFP), which binds with high affinity to *Listeria* surface carbohydrates when bacteria are cytosolic but not in a vacuole (Henry *et al*., 2006). Although not affected in actin ring formation, *prfA*^L48F^ and *prfA*^Y63W^ associated with actin tails only occasionally, consistent with their cell-to-cell-defective phenotype. No actin accumulation was observed at the time points examined for the control escape-deficient Δ*hly* and actin polymerization-deficient Δ*actA* mutants (Fig. S9).

To determine if the above observations were unique to the human epithelial cell line HeLa or generally applicable to other cells relevant to *Listeria* pathogenesis, intracellular proliferation and vacuolar escape experiments were also conducted using the mouse-derived macrophage-like cell line J774, with the same results (Fig. S11).

Collectively, these data indicate that the activation-deficient *prfA*^L48F^ and *prfA*^Y63W^ alleles confer normal ability to escape the phagosome but are unable to support efficient actin-based intracellular motility and cell-to-cell spread.

### *In vivo* survival in mouse organs

**Fig. 8:**
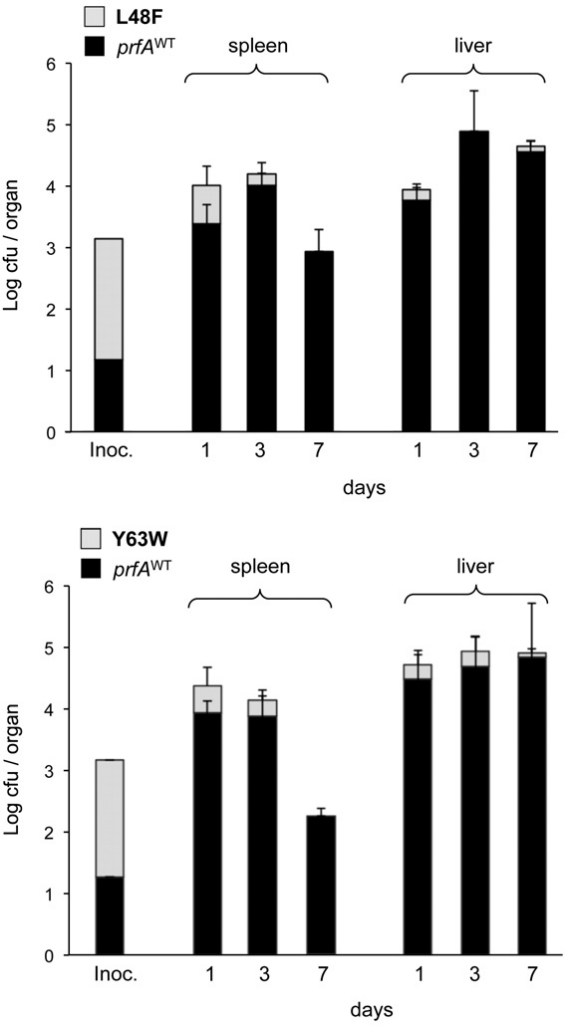
Mouse virulence tests. Competitive assay in which BALB/c mice (*n* = 9 per group) were inoculated i.v. with ≍ 10^3^ cfu of a ≍ 1:1 mixture of *prfA*^WT^ and *prfA*^allo^ mutant. Bacterial loads monitored in spleen and liver at the indicated time points. Bars represent the total cfu per organ expressed in log units with indication of the proportion of *prfA*^WT^ and *prfA*^allo^ bacteria.

Finally, we examined the ability of the activation-deficient *prfA*^L48F^ and *prfA*^Y63W^ alleles to support *in vivo* survival in a mouse model of systemic infection using a competitive virulence assay. BALB/c mice were intravenously inoculated with a ≍ 1:1 mix of mutant and *prfA*^WT^ strains and the corresponding bacterial loads monitored in the liver and spleen on days 1, 3 and 7 after infection. The *prfA*^L48F^ and *prfA*^Y63W^ strains were rapidly outcompeted by the *prfA*^WT^ strain (Fig. 8), indicating that full activation of PrfA is required for successful colonization of host tissues.

## Discussion

We report the identification of a solvent-accessible cavity within the *L. monocytogenes* master virulence regulator PrfA and provide evidence, by site-directed mutagenesis, for the involvement of this pocket in virulence gene activation during host cell infection. Two of the pocket substitutions analysed, L48F and Y63W, virtually abolished PrfA intracellular activation, consistent with the predicted phenotype for allosteric (PrfA^allo^) substitutions preventing the switching to/stabilization of a strongly active ‘ON’ state. The pocket is located inside the β-barrel that forms most of the N-terminal domain of the PrfA protomer. This domain is structurally homologous to the CNBD, an ancient signalling module present in proteins regulated by small molecules (Berman *et al*., 2005; Kannan *et al*., 2007; Kornev *et al*., 2008). The pocket occupies a similar position to the cAMP binding site of structurally related Crp but differs significantly in topology and lacks the cyclic nucleotide-anchoring motif, consistent with the reported lack of effect of cyclic nucleotides on PrfA activity (Vega *et al*., 1998). The identified pocket is different from the ‘tunnel’ proposed by Eiting *et al*. (2005) and Scortti *et al*. (2007) as the possible binding site for the putative PrfA-activating cofactor. This tunnel corresponds in fact to the interdomain cleft of PrfA, which forms a wide gap diagonally traversing the protomer (Fig. S12). One end of this tunnel in Crp serves as cAMP-binding pocket entrance, explaining the confusion. However, in PrfA the interdomain tunnel and the N-terminal pocket are unrelated since the opening of the latter is located at the opposite side of the β-barrel (Figs 2 and S12). Recently, Xayarath *et al*. (2011) reported mutagenesis analysis of two positively charged residues, K64 and K122, at each side of the ‘top’ entrance of the interdomain tunnel, and found they had only a modest contribution to PrfA activation. The K130Q substitution found in that study to abolish PrfA activity lies mostly outside the tunnel and targets an exposed residue of the C-terminal section of the central C-helix involved in inter-protomer contacts and dimer stabilization. The role of the interdomain tunnel in PrfA function remains to be determined.

### Structure–function analysis of PrfA pocket substitutions

Of the two activation-deficient substitutions identified, Y63W satisfied all the criteria of a *bona fide* PrfA^allo^ substitution, as it caused no detectable structure–function alterations in the PrfA protein that could explain the functional defect other than through largely local changes in the pocket. L48F, in contrast, impaired the intrinsic DNA-binding activity and thermal stability of PrfA. The substituting phenylalanine is predicted to create several hydrophobic clashes with neighbouring residues, probably causing re-adjustments in the structure and in the relative position of the PrfA monomers (Table S1). However, dimerization is not affected and the double L48F/G145S mutant retains the PrfA* phenotype. Thus, although introducing significant perturbations, L48F appears to preserve the general structural integrity of PrfA, consistent with its phenotype being due, partially at least, to inability to trigger an allosteric activating effect. L48F and Y63W are predicted to obstruct the entrance of the distal chamber of the pocket (Fig. S5), suggesting that their mechanism may involve steric hindrance of ligand binding. Y63W also causes the loss of a hydroxyl group that is prominently exposed at the surface of the cavity, where it may be involved in protein-ligand hydrogen bonding. Alternatively, the mechanism of these mutations may involve an ON-OFF equilibrium shift towards the OFF state in a liganded PrfA.

Two other substitutions near the distal chamber entrance, S71L and I69W, and L120V on αC at the end of the chamber (Figs 3 and S5), also significantly impaired intracellular virulence gene activation. These observations point to an important role for the innermost section of the solvent-accessible pocket in PrfA allostery. The distal chamber contacts the central C-helices that provide most of the dimer interface (Figs 2, 3 and S3) near where they tilt by 11° and straighten in the activated PrfA*^G145S^ mutant form, bringing the C-terminal ends of the C-helices towards each other (Eiting *et al*., 2005). This movement ‘tightens’ the dimer (Table S1) and is likely to be important in the conformational readjustments of the C-terminal DNA-binding domain involved in PrfA activation (Eiting *et al*., 2005). Indeed, the αC helices at the C-terminus of the CNBD in cAMP-regulated proteins are critical for long-range transmission of the allosteric signal to the effector domains (Das *et al*., 2007; Rehmann *et al*., 2007; Kornev *et al*., 2008). This mechanism involves a movement towards the cAMP-liganded pocket in the case of the shorter αC ‘lid’ of the mammalian CNBDs (Rehmann *et al*., 2007), or packing of the liganded β-barrel against the elongated inter-protomeric αC helices in the enterobacterial and mycobacterial Crp transcription factors (Popovych *et al*., 2009; Reddy *et al*., 2009). Conceivably, penetration of a ligand into the distal chamber may directly affect the relative alignment of the monomers by altering the conformation at the point where the two C-helices lie closest to each other (Fig. S3).

In the cAMP–Crp complex, a flap formed by the β4–β5 antiparallel hairpin of the β-barrel moves towards αC, tightening the liganded pocket (Popovych *et al*., 2009). A similar effect may also contribute to monomer realignment in activated PrfA, via contacts between the flap and the C-terminal end of αC′ from the opposite monomer. The two residues affected by our PrfA^allo^ substitutions, L48 and Y63, sit each on one of the two β-strands of the flap, suggesting a critical role for this structure in PrfA allosteric activation (Fig. S13).

The elevated intrinsic activity associated with F29M, I69W and S71L also supports a role for the N-terminal pocket in PrfA allostery. Basal activity is modest compared with that caused by the G145S PrfA* substitution in αD, which acts locally on the adjacent HTH motif in the C-terminal domain (Eiting *et al*., 2005). Other PrfA* substitutions in the β-roll have also previously been shown to confer weaker intrinsic activity compared with G145S, e.g. I45S (Vega *et al*., 2004) or Y63C (Miner *et al*., 2008). The partial PrfA* phenotype may be explained by these substitutions causing structural rearrangements partially mimicking ligand binding (while at the same time also interfering with PrfA allosteric activation in the case of I69W and S71L). Alternatively, these substitutions may skew the apo-PrfA equilibrium towards the ON state.

We also probed an electronegative patch at the entrance of the pocket (Fig. S4). E36 was substituted with a neutral but polar amide (glutamine) and a positively charged residue (arginine). E36Q had little or no effect but E36R resulted in ≍ 20% to 40% lower intracellular activation than wild type. The electronegative pocket entrance may therefore play some role in PrfA activation, but is unlikely to be critical. The neutrality of F29M and V80L indicates these substitutions are conformationally accommodated in the ON (intracellular) PrfA state.

### Role of PrfA allostery in *L. monocytogenes* virulence

Our data with the *prfA*^L48F^ and *prfA*^Y63W^ mutants suggest that the virulence gene induction levels achieved with PrfA in weakly active (OFF) state are sufficient for early intracellular survival/growth, whereas full PrfA activation is required for successful host tissue colonization via cell-to-cell spread. Microscopic examination of infected cells confirmed that both *prfA*^allo^ mutants were not affected in phagosomal escape but were impaired in actin tail formation. It is tempting to correlate these observations with the known differential regulation by PrfA of the primary determinants responsible for phagosomal escape and cell-to-cell spread, *hly* and *actA* respectively. Whereas the PrfA-dependent *hly* promoter has a perfectly symmetrical, high-affinity PrfA-box with a low activation threshold, *actA* is expressed from a promoter carrying a ‘mismatched’ PrfA box requiring a larger input for full induction (Scortti *et al*., 2007 and references therein). Therefore, PrfA allostery adds an additional layer of regulation acting alongside the differential response of the PrfA-regulated promoters to ensure the correct spatiotemporal expression of listerial virulence genes during infection. Our findings explain the apparent paradox between the need for escape to the cytosol for PrfA-dependent gene induction to occur (Freitag and Jacobs, 1999; our unpublished observations) and the fact that escape itself depends on PrfA-dependent determinants. The conflict is resolved very simply: while PrfA is still needed, its (allosteric) activation is not required for bacteria to access the cytosol.

Interestingly, while not forming actin tails, *L. monocytogenes prfA*^L48F^ and *prfA*^Y63W^ did not differ from *prfA*^WT^ in actin ring accumulation. Formation of actin rings (also known as ‘actin clouds’) around bacteria precedes actin-based motility and occurs soon after vacuolar escape. Rearrangement of actin rings/clouds into polar actin tails requires the prior bacterial replication in the cytosol and occurs later during intracellular infection (Tilney and Portnoy, 1989; Mounier *et al*., 1990), when PrfA-dependent gene activation is maximal (our unpublished observations). The inability of *L. monocytogenes* expressing *prfA*^allo^ mutant alleles to make this actin ring-to-tail transition suggests that the process requires the complete induction of the *actA* gene via full PrfA activation. This is consistent with the recently postulated multi-step model of ActA polarized surface distribution, critical for actin-based motility, which involves the progressive accumulation and relocation of ActA from the bacterial sides towards the poles during growth for several generations in PrfA-upregulating conditions (Rafelski and Theriot, 2006).

### Concluding remarks

Our study provides experimental support for the notion that *Listeria* virulence depends on the intracellular activation of PrfA via an allosteric pocket in its N-terminal domain. The shape and physicochemical characteristics of the pocket suggest it could bind a relatively hydrophobic, elongated molecule with an aromatic moiety lodging in the inner chamber. Confirmation of the role of this pocket in PrfA allostery will require the identification of the PrfA-activating molecule and co-crystallization studies. The putative allosteric effector may be an endogenous bacterial metabolite generated upon sensing the host cell habitat or, alternatively, a host-derived molecule imported into *L. monocytogenes* during intracellular infection. This molecule may form the basis for PrfA-inhibitory analogues potentially useful in anti-*Listeria* chemotherapy.

## Experimental procedures

### Bacterial strains, plasmids, culture conditions and chemicals

The bacteria and plasmids used in this study are listed in Table S2. *L. monocytogenes* and *E. coli* were grown in BHI and Luria–Bertani (LB) base media, respectively, supplemented with antibiotics as appropriate to ensure plasmid maintenance (7.5 µg ml^−1^ for pPL2 integrants in *Listeria*, 15 µg ml^−1^ chloramphenicol and 50 µg ml^−1^ kanamycin for pPL2 and pET28a derivatives in *E. coli*). Semi-quantitative detection of PrfA-dependent gene expression using the *L. monocytogenes plcB* gene as a reporter (*plcB* is transcribed from the P*actA* promoter; Vazquez-Boland *et al*., 1992) was carried out on egg yolk BHI plates as previously described (Ermolaeva *et al*., 2004). All incubations were performed at 37°C. Chemicals were purchased from Sigma-Aldrich unless stated otherwise.

### General DNA techniques

Restriction enzymes were used according to the manufacturers' instructions (Promega and New England Biolabs). Plasmid DNA was extracted from *E. coli* using the plasmid purification kit from Qiagen. PCR was carried out using Biotools *Taq* DNA polymerase or high-fidelity PfuUltra II Hotstart DNA Polymerase (Agilent) for *prfA* gene constructions. PCR products were purified with the PCR purification kit from Qiagen. Sanger DNA sequencing was carried out on both strands on an ABI3700 instrument (Applied Biosystems).

### Construction of site-directed *prfA* mutants

Codon mutations were introduced into the *prfA* gene by overlap extension (Ho *et al*., 1989). The two intermediate PCR products with overlapping 3′ ends were generated with oligonucleotide primers external to *prfA* (MR2KpnI, MR10SpeI) and appropriate forward and reverse internal primers harbouring the relevant mutations (Table S3). The fusion PCR products carrying the different *prfA* constructs were inserted into the integrative vector pPL2 (Lauer *et al*., 2002) using the SpeI and KpnI restriction sites of the external primers. The plasmids were introduced into *L. monocytogenes*Δ*prfA* by electroporation and integrants were selected by plating onto BHI containing 7.5 µg ml^−1^ chloramphenicol. Plasmid integration at the correct place was checked by PCR and the inserted *prfA* allele by DNA sequencing.

### *L. monocytogenes* cell-free extracts, SDS-PAGE and Western immunoblotting

Bacteria grown to OD_600_ = 1.0 were lysed as described for recombinant PrfA purification. Total protein was determined with the colorimetric DC protein assay (Bio-Rad). SDS-PAGE was carried out on 15% acrylamide separation gels and anti-PrfA Western immunoblotting was performed as previously described (Ermolaeva *et al*., 2004) using polyvinylidene difluoride (PVDF) membranes and Amersham ECL chemiluminiscent detection reagents (GE Healthcare).

### Transcription analysis by RT-QPCR

Total RNA was extracted by mixing 1 ml of RNAprotect Bacteria Reagent (Qiagen) with 500 µl of *Listeria* mid-exponential-phase BHI cultures (OD_600_ = 0.3) or suspensions of infected HeLa cells (see below) in PBS. The mixtures were incubated for 5 min at room temperature, centrifuged at 5000 *g* for 10 min at 4°C and stored at −80°C until use. Frozen pellets were resuspended in Buffer RA1 (Macherey-Nagel) containing 1% β-mercaptoethanol, transferred to Lysis Matrix B tubes containing 0.1 mm silica beads (Q-Biogene) and homogenized in a FastPrep instrument (Q-Biogene) for 40 s at speed 6. After centrifugation at 12 000 *g* for 5 min at 4°C, the supernatants were collected and the nucleic acids precipitated with an equal volume of ethanol. The RNA was purified with a Nucleospin RNA II kit (Macherey-Nagel), treated with 10 U DNase I (Promega) for 30 min at 37°C, repurified using NucleoSpin® RNAII, and eluted in 50 µl of RNase- and DNase-free water (Promega). The first-strand cDNA was synthesized in 20 µl reaction volumes using 5 µl of total RNA, 100 µM random hexamers (Eurogentec, Belgium) and the ImProm-II™ Reverse Transcription System (Promega). The cDNA samples were diluted 1:10 in nuclease-free water and quantitative real-time PCR was performed in 20 µl reaction volumes containing 1× PCR TaqMan buffer II, 6 mM MgCl_2_, 200 µM dNTP, 300 nM primers, 100 nM or 150 nM of the probe (Table S3), 1 U of AmpliTaq Gold DNA polymerase (Applied Biosystems) and 5 µl of cDNA preparation. Reactions were run on a StepOnePlus System (Applied Biosystems) using the following programme: 10 min at 95°C and 40 cycles of 15 s at 95°C and 1 min at 60°C. All samples were amplified in duplicate and threshold cycle (C_T_) values ≥ 40 were considered negative. Expression data were normalized by calculating the ratio between the number of transcripts of the target gene and those of the *L. monocytogenes* housekeeping genes *ldh* and *rpoB*. Both reference genes were previously demonstrated to be constitutively expressed in our experimental conditions.

### Recombinant PrfA protein production and purification

PrfA proteins were produced in *E. coli* using the pET28a expression vector (Novagen). *prfA* alleles were amplified by PCR from the pPL2 plasmid constructs (Table S2) using the oligonucleotide primer pair prfAH1-P14 and prfAH2-P14, which contain NdeI and SalI restriction sites respectively (Table S3). The *prfA*-containing amplicons were then inserted into pET28a using the NdeI and SalI sites and the pET28a*prfA* constructs were introduced into *E. coli* Bl21(DE_3_) by electroporation. Recombinant *E. coli* strains were grown in 500 ml of LB broth until OD_600_ = 0.8, then induced by adding 1 mM IPTG and incubating for 3 h. The bacteria were pelleted by centrifugation and resuspended in 500 µl of lysis buffer (50 mM NaH_2_PO_4_, 300 mM NaCl, 20 mM imidazole) containing Protease Inhibitor Cocktail (Roche). The bacterial suspensions were incubated for 30 min on ice with 1 mg ml^−1^ lysozyme, transferred to Lysing Matrix B tubes and subjected to three 30 s rounds of FastPrep homogenization at speed 6.5. Cell debris was removed by centrifugation (10 000 *g*, 20 min, 4°C) and the N-terminal His-tagged recombinant PrfA proteins were purified from the bacterial soluble extract by affinity chromatography on a HiTrap IMAC FF nickel column in an AKTA system (GE Healthcare). Fractions containing the purified PrfA proteins were pooled and stored at −20°C in elution buffer with 20% glycerol.

### Biophysical characterization of PrfA proteins

Equilibrium sedimentation was carried out at 60 000 r.p.m. for 3 h at 22°C in a Beckman Optima XL-I ultracentrifuge using protein samples of 400 µl with an OD_280_ = 0–8 to 1.0, determined in a NanoDrop microspectrometer (Thermo Scientific). Prior to the experiments, the buffer of purified PrfA stocks was changed to 10 mM HEPES pH 7.4, 150 mM NaCl, 3 mM EDTA, 1 mM TCEP and 10% glycerol. Data were processed using Sedfit Analysis Software v1180. The molecular mass in Daltons was derived from the ordinate maximum of the molar mass distribution coefficient *c*(*M*).

For fluorescence-based thermal stability assays, samples of 25 µl containing 3.5 µg of purified recombinant PrfA protein and 5 µl of 12.5× Sypro Orange solution (Invitrogen) diluted 1/400 in the protein buffer were gradually heated in an IQ5 96-well format real-time PCR instrument (Bio-Rad) to a range of temperatures between 25°C and 95°C with a heating rate of 0.5°C min^−1^. Fluorescence intensity was measured every min at excitation and emission wavelengths of 470 and 570 nm, respectively. The inflection point of the melting transition from folded and unfolded states (melting temperature, *T*_m_) was determined from the first derivative of the plot of fluorescence intensities (Lo *et al*., 2004). Shifts in the *T*_m_ reflect differences in stability associated with substitution-induced conformational changes (Ericsson *et al*., 2006).

The DNA-binding affinity of purified PrfA proteins was determined using a Biacore biosensor system T100. Biotinylated oligonucleotides (40 bp) containing the PrfA box of *plcA*/*hly* or *actA* promoters in a central position (Table S3) were immobilized on a streptavidin-coated sensor chip (SA Chip, Amersham-Pharmacia Biotech). To create double-stranded DNA, saturating amounts of non-biotinylated complementary DNA were flowed over the chip. Binding assays were performed at 25°C in 10 mM HEPES pH 7.4, 150 mM NaCl, 3 mM EDTA, 0.005% v/v P20 surfactant (HBS-EP buffer, Biacore Life Sciences) containing 1 mM Tris(2-carboxyethyl)phosphine. PrfA proteins were injected at concentrations in the range of 0.3 nM to 6 µM at a flow rate of 75 µl min^−1^. DNA binding was measured for 240 s until binding approached a steady state, followed by dissociation for another 120 s and surface regeneration with 0.1% SDS 3 mM EDTA for 60 s at 100 µl min^−1^. The reference signal (oligonucleotide-free streptavidin-coated cell) was subtracted from each sensorgram and the resulting curves were aligned to a common baseline. Some experiments included a flow cell coated with a random 40-mer oligonucleotide; no significant binding being detected in these controls. For kinetic constants and binding affinity determinations, the 1:1 Langmuir binding model was used as the fitting model in the Biacore T100 Evaluation software. The fits showed chi-square values of between 1 and 10.

### Structural analyses and modelling

Theoretical models of the structures of PrfA mutants were obtained using modeller (Sali and Blundell, 1993), which generates protein structures by satisfaction of spatial restraints with simultaneous optimization of charmm energies, conjugate gradients and molecular dynamics with simulated annealing. Comparative models were validated with prochek (Laskowski *et al*., 1993), what_check (Hooft *et al*., 1996), verify3d (Luthy *et al*., 1992) and joy (Mizuguchi *et al*., 1998). The crystal structure of PrfA^WT^ at 2.7 Å resolution (PDB code 2BEO, Eiting *et al*., 2005) was used as the template for modelling the structures of the PrfA mutants. The interactions between the new side-chains and the surrounding residues, and the accessible surface areas (ASA), gap volumes and gap volume indexes were calculated from the models of the PrfA mutants to infer possible changes in the relative positions of the two protomers of PrfA (Table S1). Dimer interface surfaces were calculated as variations of the ASAs on complexation (Jones and Thornton, 1996). Gap volumes between the two chains of the PrfA dimer were calculated using the program surfnet (Laskowski, 1995). Interface ASA, gap volume and gap volume index values were obtained from the server protorp (Reynolds *et al*., 2009). Electrostatic interactions between charged residues, and contact atoms and type of interactions were identified using the in-house programs elecint and contacts (R. Núñez Miguel, unpublished). Structural alignment by co-ordinate superposition was carried out using mnyfit (Sutcliffe *et al*., 1987). The graphical program MacPyMOL (DeLano Scientific LLC, http://www.pymol.org) was used for visualization and preparation of figures of protein structures.

### Mammalian cell cultures and infection experiments

Low-passage cell lines were grown in 60 mm tissue culture dishes (RT-QPCR assays) or 24-well plates (intracellular proliferation assays) at 37°C under 5% CO_2_ in DMEM without antibiotics supplemented with 10% fetal bovine serum (FBS) and 2 mM l-glutamine (Biowhittaker) until 90–100% confluence. *L. monocytogenes* inocula were prepared from BHI cultures grown until OD_600_ = 1.0. Bacterial cells were washed three times in PBS and stored at −80°C in 20% glycerol PBS. HeLa cells were inoculated at a multiplicity of infection (moi) carefully adjusted according to the invasiveness of the strain so as to obtain similar numbers of intracellular bacteria at *t* = 0 (moi ranging from 1:1 for hyperinvasive prfA*^G145S^ bacteria to 150–170:1 for non-invasive Δ*prfA* bacteria). J774A.1 macrophages were infected using an moi of 1:1. Immediately after infection, cell monolayers were centrifuged for 3 min at 172 *g* at room temperature, incubated for 15 min (J774A.1 cells) or 40 min (HeLa cells), washed twice with Dulbecco's PBS (DPBS, Gibco) to remove non-adherent bacteria, and incubated in DMEM supplemented with gentamicin (100 µg ml^−1^ during 1 h and 10 µg ml^−1^ thereafter) to prevent extracellular bacterial growth. Infected cells were washed twice and resuspended in 400 or 700 µl of PBS for plate counting or RNA extraction, respectively. Since the intracellular bacterial population at a given time point depends on the initial number of bacteria that successfully invaded the cell monolayer, intracellular proliferation data were normalized using an ‘Intracellular Growth Coefficient’ according to the formula IGC = (IB*_t_*_=_*_n_* − IB*_t_*_=0_)/IB*_t_*_=0_ where IB*_t_*_=_*_n_* and IB*_t_*_=0_ are the intracellular bacterial numbers at a specific time point *n* and *t* = 0 respectively.

Plaque assays were carried out according to Sun *et al*. (1990), with modifications. L929 fibroblasts were grown and infected as described above except that six-well plates (Costar), RPMI medium and moi of 0.005:1 to 2:1 were used. After 1 h incubation with 50 µg ml^−1^ gentamicin, infected monolayers were washed three times with DPBS and overlaid with 1.5 ml of melted RPMI containing 1% cell culture grade agar and 5 µg ml^−1^ gentamicin. Plaques were visualized after 4-day incubation by staining with 10% Neutral Red solution in DPBS. Diameters of a minimum of 20 randomly selected plaques per well were measured on digital images.

### Phagosomal escape assays

The ability of *Listeria* strains to escape from the phagocytic vacuole was assessed using two separate fluorescence microscopy-based assays. HeLa cells were grown on 13 mm coverslips in 24-well plates as described above. For the first assay, 5.0 × 10^4^ HeLa cells were seeded in each well, incubated 24 h, infected at 50:1 moi and sampled after 10, 20, 30, 45 and 90 min. At each time point, infected monolayers were washed four times in warm PBS to remove extracellular bacteria except for *t* = 45 and 90 min, in which 100 µg ml^−1^ gentamicin was added after 30 min of infection to prevent extracellular bacterial growth. Experiments with J774A.1 macrophages were performed identically, except that 1.0 × 10^5^ cells per well and an moi of 10:1 were used. Coverslips were fixed with 3.7% (w/v) paraformaldehyde and prepared for microscopic examination by permeabilization with 0.2% (v/v) Triton X-100 in PBS for 15 min, blocking with 3% (w/v) bovine serum albumin (BSA) in PBS for 1 h, and a further incubation of 1 h with primary antibody to Rab7 (Cell Signaling) in the same buffer. This was followed by 1 h incubation with AlexaFluor 568-conjugated anti-rabbit IgG secondary antibody (Invitrogen) and 20 min incubation with AlexaFluor 488-conjugated phalloidin (Invitrogen) and DAPI to visualize F-actin and bacterial DNA/cell nuclei, respectively. Coverslips were mounted using ProLong Anti-fade reagent (Invitrogen). Images were acquired using a Leica CTR-6000 immunofluorescence microscope. Quantification of vacuolar and cytosolic *Listeria* was performed by counting the proportion of bacteria associated with either the Rab7 endosomal marker or F-actin, respectively, per image field.

For the second assay, 3.5 × 10^4^ HeLa cells were seeded per well 24 h prior to transfection with the mammalian expression plasmid pEYFP-C1-CBD (Henry *et al*., 2006) using Lipofectamine-2000 reagent (Invitrogen) according to the manufacturer's instructions. Briefly, Lipofectamine-2000 and plasmid DNA were diluted in opti-MEM at 1:100 or a concentration of 10 µg ml^−1^, respectively, and equal volumes of each solution were mixed. After incubation for 30 min the liposome–plasmid complexes were added to the cells followed by 24 h incubation. Transfection efficiency was typically > 85%. Transfected cells were infected with *Listeria* strains, time points taken, and coverslips processed and stained with AlexaFluor 546-conjugated phalloidin and DAPI as described above. Upon microscopic examination, ring-like accumulations of the YFP probe (and/or F-actin) around the bacteria indicated presence in the cytosol.

### Mouse virulence assay

Groups of 6- to 8-week-old female BALB/c mice were infected via the tail vein with 2 × 10^3^ cfu of a ≍ 1:1 mix of *prfA*^WT^ and *prfA*^allo^*L. monocytogenes* bacteria. After euthanasia at days 1, 3 and 7 after infection, livers and spleens were recovered, homogenized and the bacterial loads determined by plate counting (three mice per group per time point). At least 20 colonies per time point and animal were randomly analysed to determine the proportion of *prfA*^WT^ and *prfA*^allo^ bacteria by PCR, based on the opposite orientation of the constructs in the integration vector (primers PrfA-3F, PrfA-T3 and PrfA-2R; Table S3). Animals were bred and maintained in pathogen-free conditions at the School of Biological Sciences Animal Facility, University of Edinburgh (UK). Experiments were covered by a Project Licence granted by the UK Home Office under the 1986 Animals (Scientific Procedures). The University of Edinburgh Ethical Review Committee approved this licence and the experiments.

### Statistics

The statistical significance of data was assessed by paired Student's *t*-tests using PASW statistic 17.0 software (SPSS, Chicago, IL).
