# Supplementary material for: Allosteric mutants show that PrfA activation is dispensable for vacuole escape but required for efficient spread and Listeria survival in vivo
Source: Mol Microbiol. 2012 Aug;85(3):461–77. doi: 10.1111/j.1365-2958.2012.08121.x (PMC3443378; doi:10.1111/j.1365-2958.2012.08121.x)
Supplement: Supplementary file 1 [file mmi0085-0461-SD1.pdf]

Allosteric mutants show that PrfA activation is dispensable for vacuole escape but required for efficient spread and *Listeria* survival *in vivo*

Caroline Deshayes [1¶], Magdalena K. Bielecka [1¶], Robert J. Cain [1], Mariela Scortti [1,2], Aitor de las Heras [1], Zbigniew Pietras [3], Ben F. Luisi [3], Ricardo Núñez Miguel [3§], and José A. Vázquez-Boland [1,4] *

[1] Microbial Pathogenesis Unit, Centre for Infectious Diseases, University of Edinburgh, Edinburgh, UK,
[2] Departamento de Bioquímica y Biología Molecular IV, Universidad Complutense, Madrid, Spain,
[3] Department of Biochemistry, University of Cambridge, Tennis Court Road, Cambridge, UK,
[4] Grupo de Patogenómica Bacteriana, Universidad de León, León, Spain.

* E-mail v.boland@ed.ac.uk; Tel +44 (0)131 651 3619.

ONLINE SUPPORTING INFORMATION

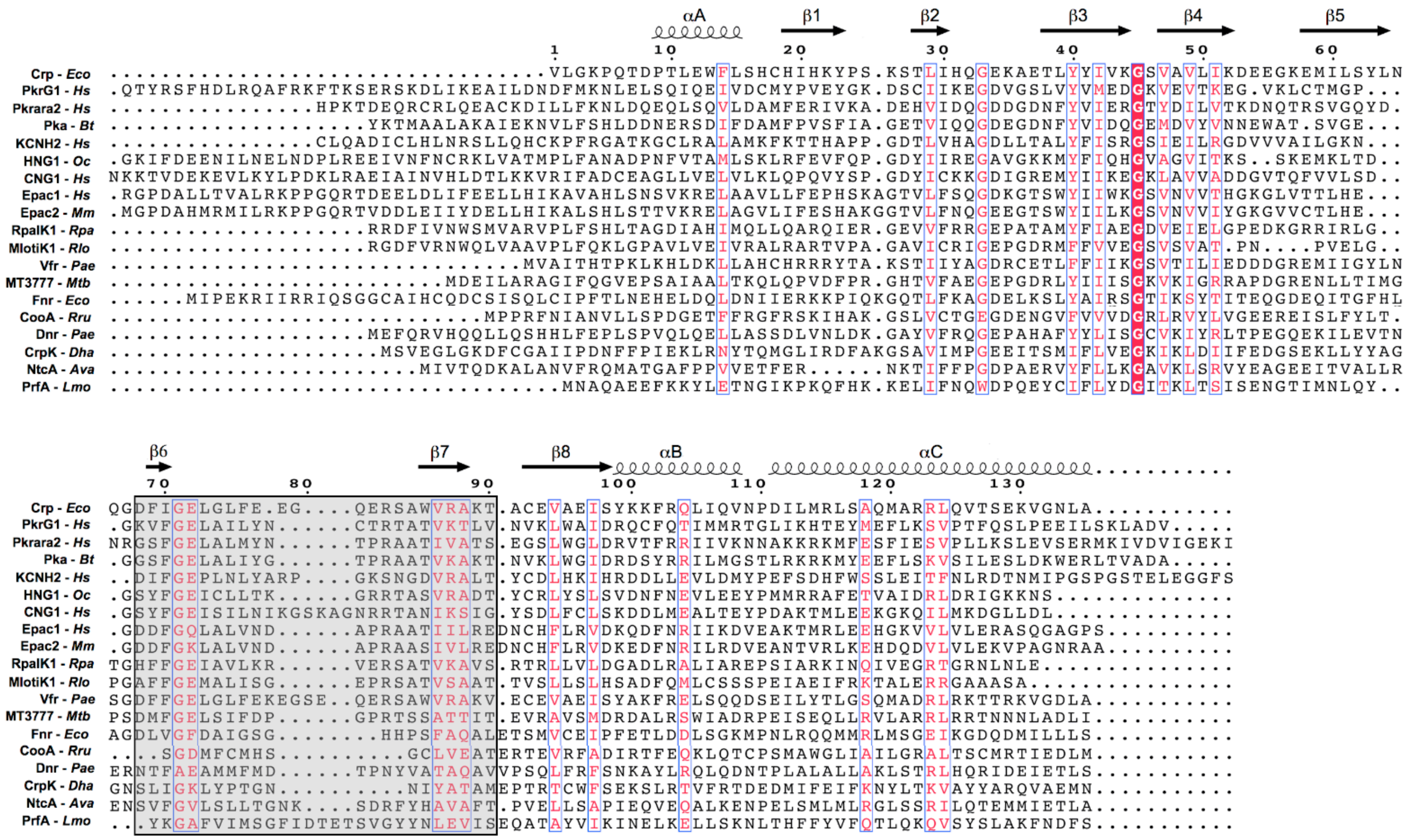

Fig. S1. Amino acid sequence alignment of CNBDs from a selection of bacterial and eukaryotic proteins. The grey-shaded box corresponds to the PBC region alignments shown in Fig. 1. Position of α-helices and β-strands of the CNBD β-barrel are indicated. Conserved blocks of sequence are shown in red case, conserved residues are shaded in red. ClustalW2 alignment (http://www.ebi.ac.uk/Tools/clustalw2/) visualised with ESPript (http://espript.ibcp.fr/; Gouet *et al.*, 1999).

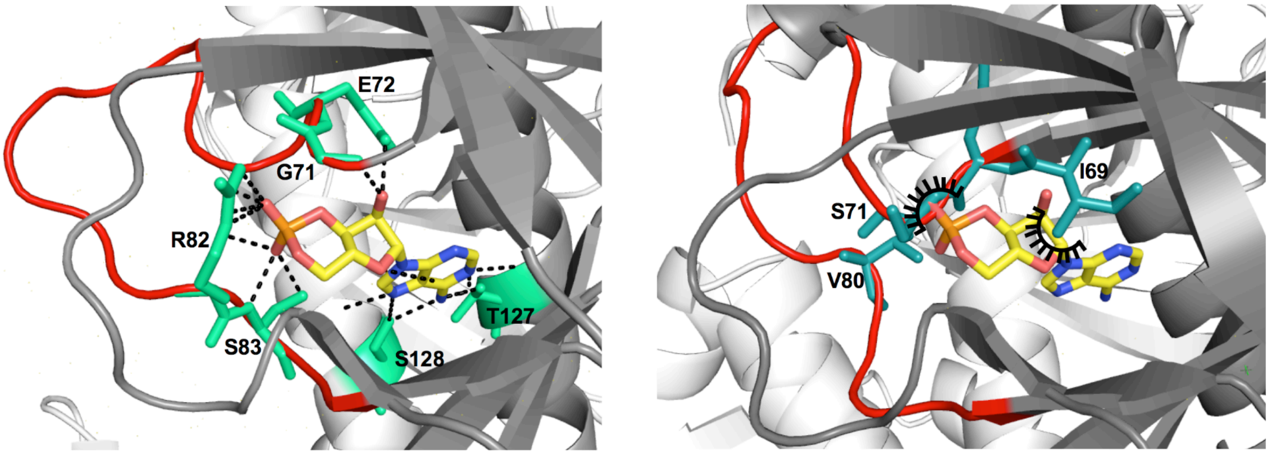

Crp **PrfA**

Fig. S2. Identification of a putative ligand-binding site in the PrfA β-barrel. Left panel, crystal structure of holo-Crp (PDB code 1G6N) showing polar contacts between cAMP molecule and residues in its binding pocket (black dashed lines). Right panel, equivalent image of PrfA^{WT} (PDB code 2BEO) with cAMP positioned in the β-barrel according to a structural alignment with the cAMP-Crp complex. The superimposed cAMP molecule lodges in a cavity in PrfA's CNBD at a similar position to Crp. In the two panels, one monomer is coloured light grey and the other dark grey. The PBC loop (and corresponding region in PrfA) is represented in red. Residues that interact (Crp) or clash (PrfA) with cAMP are shown in sticks, steric clashes are indicated with the symbol .

A

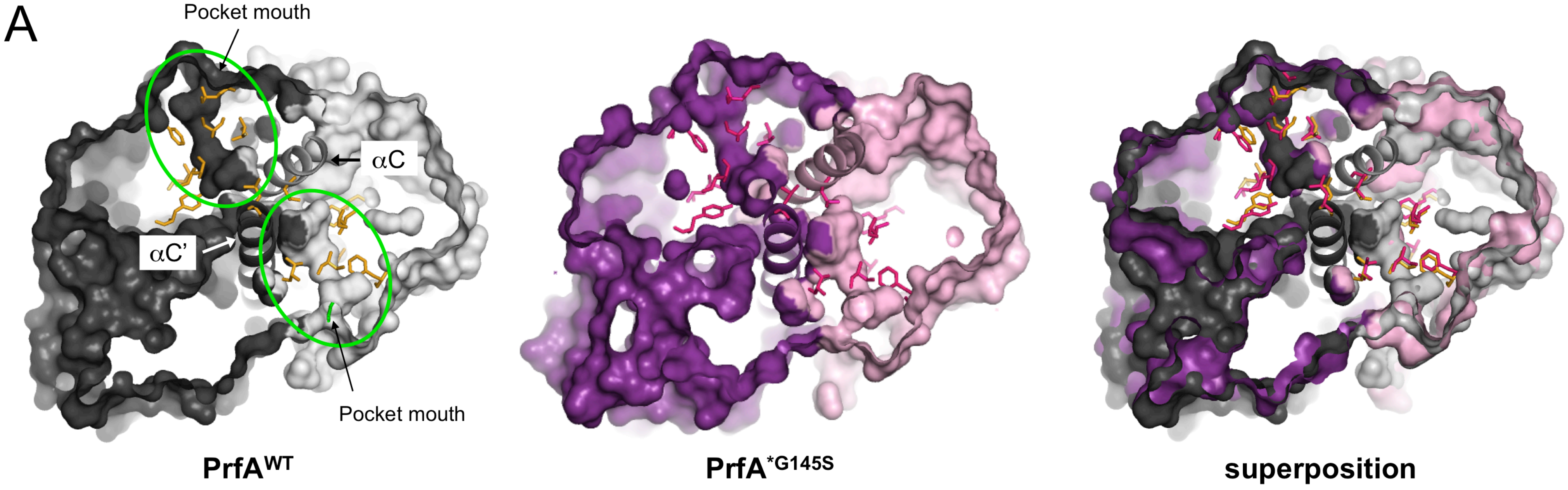

B

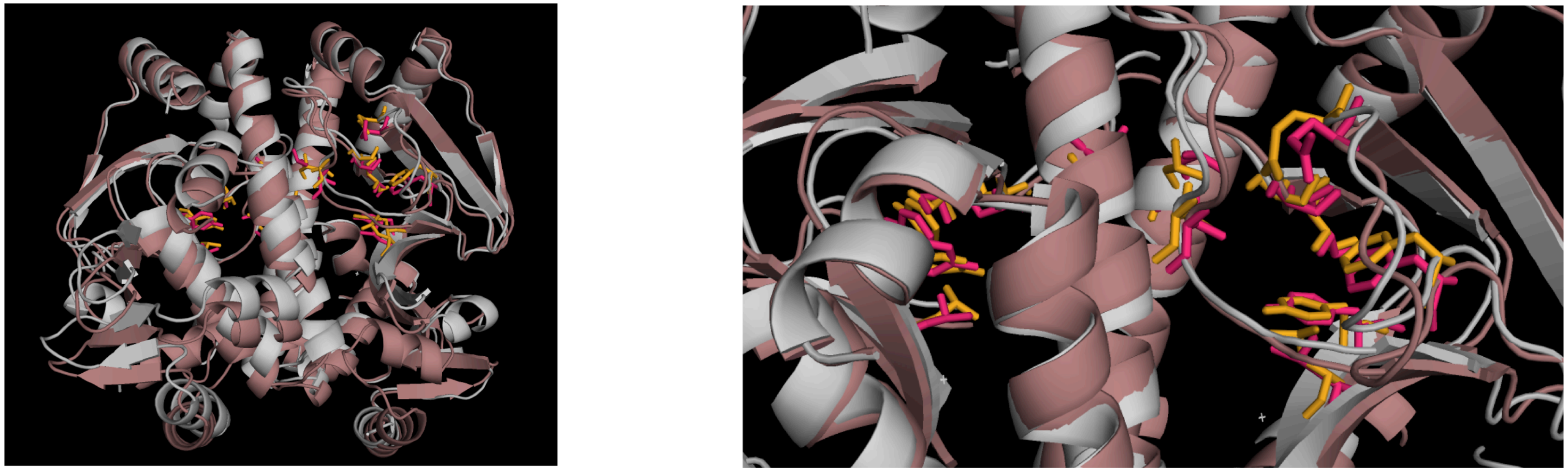

Fig. S3 (legend on next page).

Fig. S3. Visualisation of the targeted pocket residues in the crystal structures of $PrfA^{WT}$ (PDB code 2BEO) and $PrfA^{*G145S}$ (PDB code 2BGC). The pocket environment is minimally affected by the G145S PrfA* substitution in αD (see Fig. S13), which causes constitutive activation of PrfA via local structural changes in the C-terminal DNA-binding domain directly affecting the HTH motif.

A. Surface representation of the PrfA dimer ("top" view, see Fig. 2) with front and back planes clipped to show the surface contour of the N-terminal domain pockets (encircled in green on the left panel). In $PrfA^{WT}$ one protomer is coloured light grey and the other dark grey, with mutated residues in orange; in $PrfA^{*G145S}$ the protomers are violet and light pink with mutated residues in bright pink. C-helices are shown in cartoon representation (αC and αC' from the opposite monomer). The entrance of the pocket from each protomer is indicated in $PrfA^{WT}$.

B. Structural alignment of $PrfA^{WT}$ (grey) and $PrfA^{*G145S}$ (dark salmon) in ribbon representation with mutated pocket residues shown as sticks coloured as in (A). Note the minimal effects of the G145S substitution in the C-terminal domain on the relative positions of the pocket residues in the N-terminal domain (see Fig. S7).

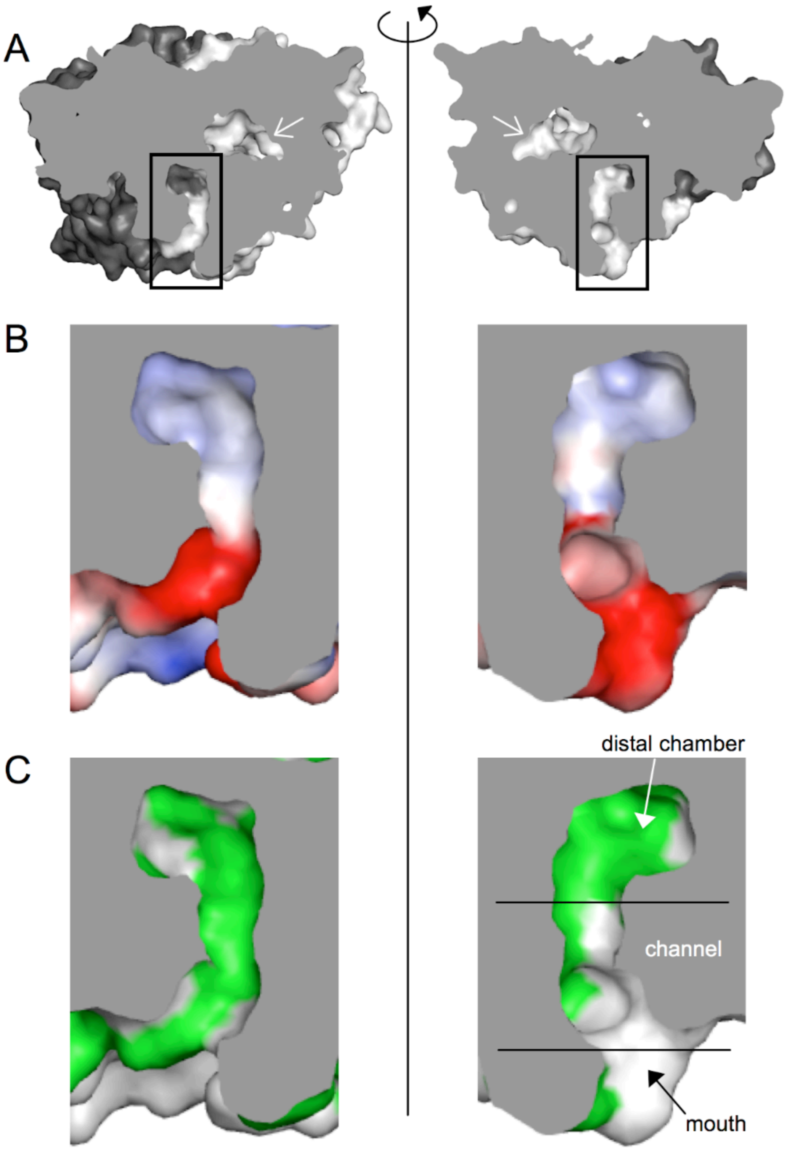

Fig. S4. PrfA pocket topology and surface properties.

A. Cutaway cross-section of the PrfA dimer ("top" view, see Fig. 2) with one half rotated 180º showing both sides of the internal surface of one of the pockets (framed in black). Arrows indicate the interdomain tunnel (see text and Fig. S12). The surfaces of one monomer are light grey, those of the opposite monomer are dark grey and the bisecting plane in flat grey.

B. Enlargement of the framed area in (A) showing qualitative surface electrostatics of the PrfA pocket, as determined with the vacuum electrostatics MacPyMOL function. Electronegative surfaces are in red, electropositive surfaces in blue.

C. Enlargement of the framed area in (A) showing the solvent-exposed hydrophobic surface area of the PrfA pocket. Hydrophobic residues (Val, Ile, Leu, Met, Phe, Tyr and Trp) are shown in green.

.

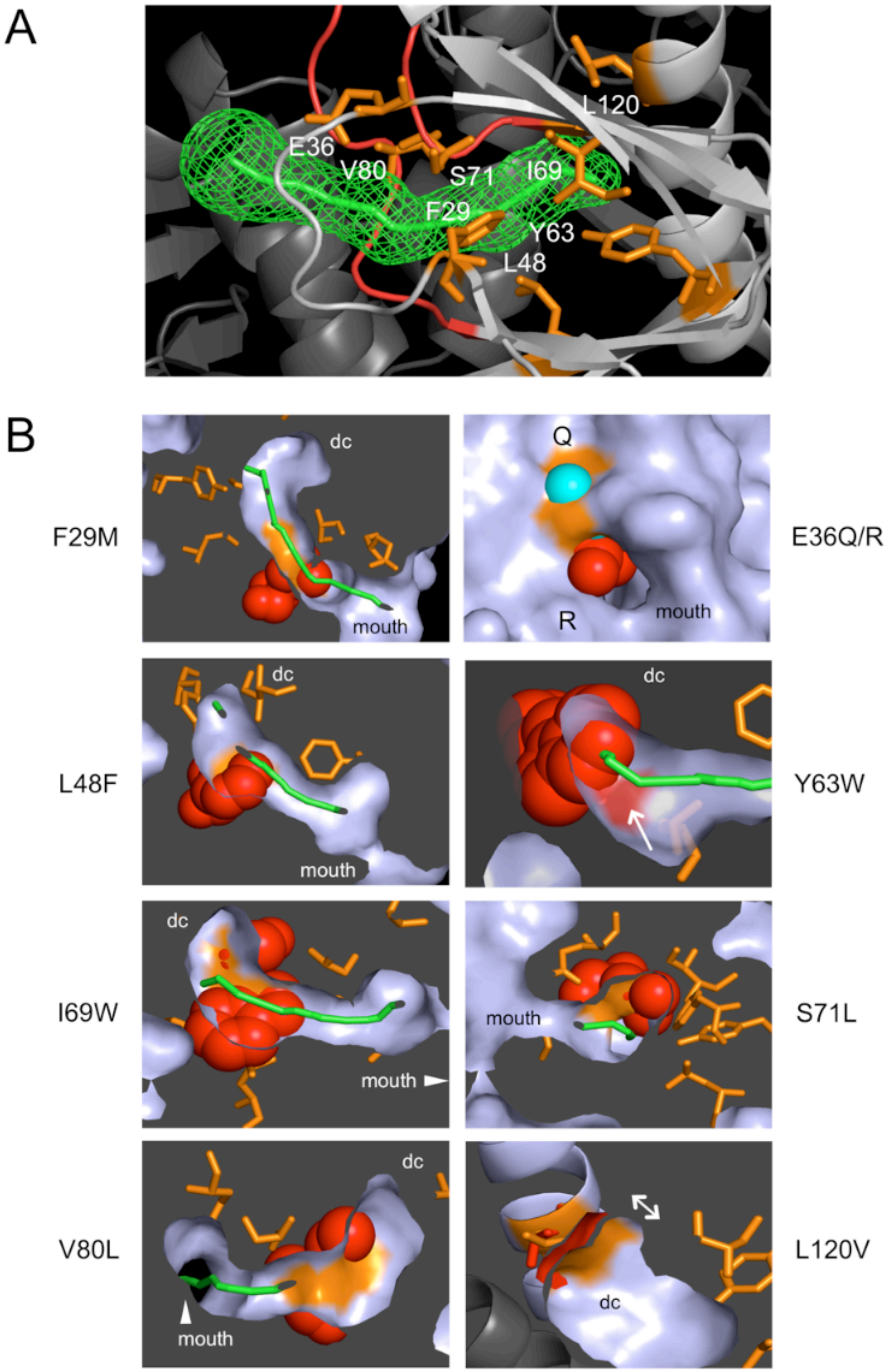

Fig. S5 (legend on next page).

Fig. S5. Site-directed mutagenesis of the N-terminal domain pocket of PrfA.

A. Close-up view of the PrfA β-barrel in cartoon format with the solvent trajectory of the internal pocket determined by CAVER (Petrek *et al.*, 2006) shown as a green mesh. The eight residues mutated in this study are represented as orange sticks (see also Fig. 3) in one of the protomers (coloured light grey; the opposite protomer is dark grey). The loop between β-strands 6 and 7 that corresponds to the PBC in Crp, and which forms one side of the mouth of the pocket in PrfA, is shown in red (see Fig. 2).

B. Modelling of the local structural alterations caused by the PrfA pocket substitutions. A cutaway section of an appropriately positioned PrfA structure in surface representation exposes the relevant portion of the internal cavity (clipping plane is in flat dark grey). In each panel, the atoms of the substituting residue are displayed in red space-fill representation (except for Q36, shown as blue spheres, and V120 represented as red sticks with the corresponding surface also in red); all other visible residues targeted in this study are shown as orange sticks. The surface of the original wild-type residue is shown in orange. The centreline of the solvent-accessible route of the pocket is shown in green. "dc" indicates the position of the distal chamber. The substitutions target residues in the proximal (F29M), middle (S71L) and distal portions (L48F, Y63W, I69W and V80L) of the channel and at the end of the pocket (L120V) plus a negatively charged patch at the pocket entrance (E36Q/R). In some cases, the substitution chosen was predicted to accommodate an infrequently observed rotameric state that would protrude into the pocket to avoid steric clashes with neighbouring residues. In E36R, a rotamer of the new arginine, besides altering the charge, also introduces a bulkier side chain that partially occludes the entrance. In L48F, Y63W, I69W and S71L, the new larger side chains are predicted to partially obstruct the internal cavity, at the entrance of the distal chamber or access channel. In the Y63W panel, the arrow indicates the position of the hydroxyl group of the Y63 side chain, which is directly exposed to the pocket surface (semitransparent red patch) and may establish polar interactions with a putative ligand (see text). The double arrow in the L120V panel indicates the predicted inward displacement of the distal cul-de-sac of the pocket against the dimer interface as a result of the shorter valine residue. L120 is on αC at the monomer-monomer interface and forms part of the hydrophobic surface of the distal chamber. Valine has a β-branched side chain with restricted rotamer choice on a helix and substitution to this shorter residue is expected to affect the shape of the pocket without altering the position of the helix. F29M was anticipated to change the shape of the wall of the pocket at a constriction in the middle of the channel. V80 occupies a dominating part of the hydrophobic surface of the distal chamber; Leucine was chosen to satisfy the criteria that the substitution produces little perturbation of the structure while still being capable of modifying the pocket shape. The lack of effect of F29M and V80L in PrfA intracellular activation indicates that these substitutions are perfectly accommodated in the "ON" (liganded) PrfA conformation. They also suggest that contacts with a hypothetical ligand are unlikely to be dominated by aromatic stacking interactions on the surface of F29.

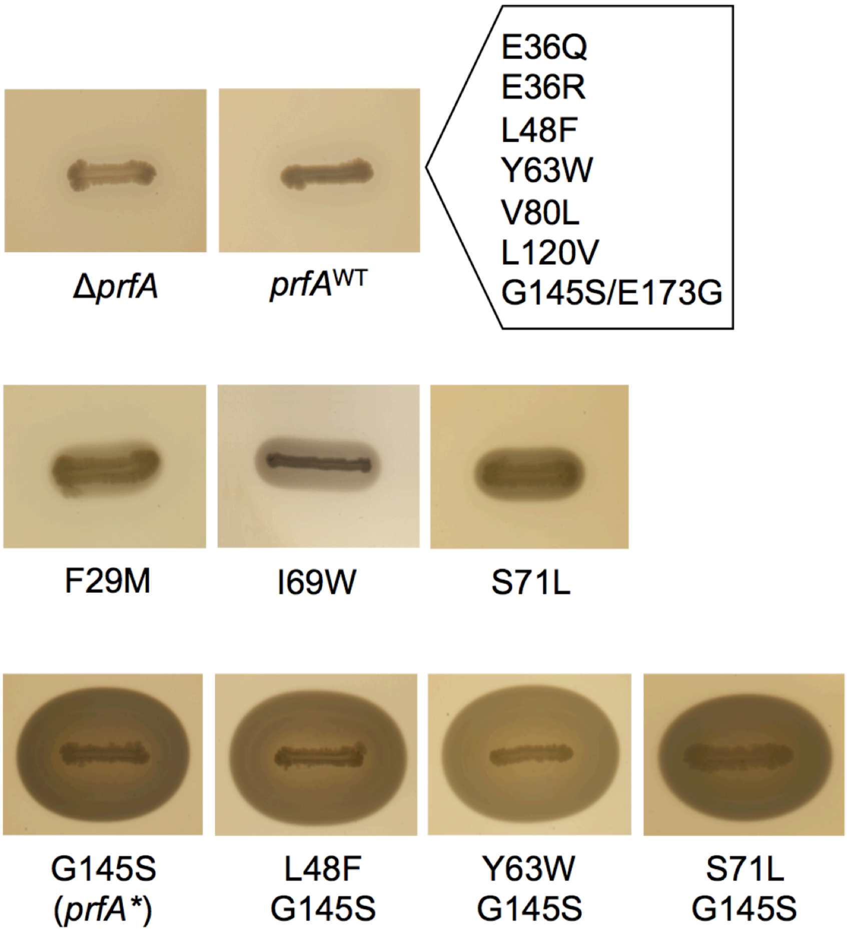

Fig. S6. Phenotype of the PrfA pocket mutants on egg yolk agar. The assay tests semiquantitatively the levels of PrfA-dependent expression using the *plcB* gene as a reporter (Ripio *et al.*, 1996). The *plcB*-encoded broad substrate range phospholipase C (PC-PLC, PlcB) causes a halo of fatty acid precipitation around the colonies that is proportional to the amount of enzyme expressed by the bacteria. Pictures taken from representative plates incubated at 37° C for 72 h. Due to the weak intrinsic activity of PrfA, PrfA-dependent expression is low and PlcB activity is undetectable in BHI at 37º C in *L. monocytogenes* carrying the $prfA^{WT}$ allele (Ripio *et al.*, 1996, 1997). The PrfA mutations boxed show the same PlcB-negative phenotype of Δ*prfA* complemented with $prfA^{WT}$. Bacteria carrying the PrfA-activating G145S substitution exhibit a PrfA* phenotype characterised by a strong, wide precipitation halo due to *plcB* overexpression. Note the partial PrfA* phenotype associated with F29M, I69W and S71L pocket substitutions, which cause a weak increase in the basal (intrinsic) DNA-binding activity of PrfA (Table 1).

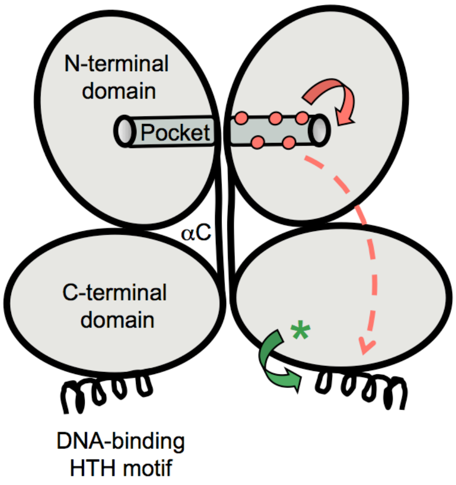

Fig. S7. A schematic of the PrfA dimer illustrating the N-terminal pocket/C-terminal PrfA*^{G145S} double mutant approach. N-terminal pocket substitutions are represented as red dots, the PrfA*^{G145S} mutation as a green asterisk. Structural changes caused by *bona fide* allosteric pocket substitutions are expected to be mainly confined to the N-terminal β-barrel (solid red arrow) and hence largely dissociable from those caused locally in the C-terminal DNA-binding domain by the PrfA-activating G145S substitution (solid green arrow). This seems to be the case, since upon introduction of activation-defective pocket substitutions in a G145S context, the strong PrfA* phenotype is retained (Figs. 4 and S6) and the corresponding proteins show strongly elevated intrinsic DNA-binding activity (Table 1). The basal activity levels, however, are lower in the double mutants compared to the single *prfA**^{G145S} mutant (Fig. 4, Table 1). This may reflect two situations: (i) in the compact PrfA molecule, the N-terminal pocket substitutions transmit some structural perturbations to the C-terminal DNA-binding domain (red broken arrow); (ii) alternatively, if the pocket and G145S substitutions act by shifting in opposite directions the "OFF-ON equilibrium" in the PrfA population, the combination of the two mutations will confer a mixed phenotype. Regardless of the intimate underlying mechanism, the double mutant strategy shows that the pocket mutations do not cause grossly disruptive damage to the PrfA fold. ñ

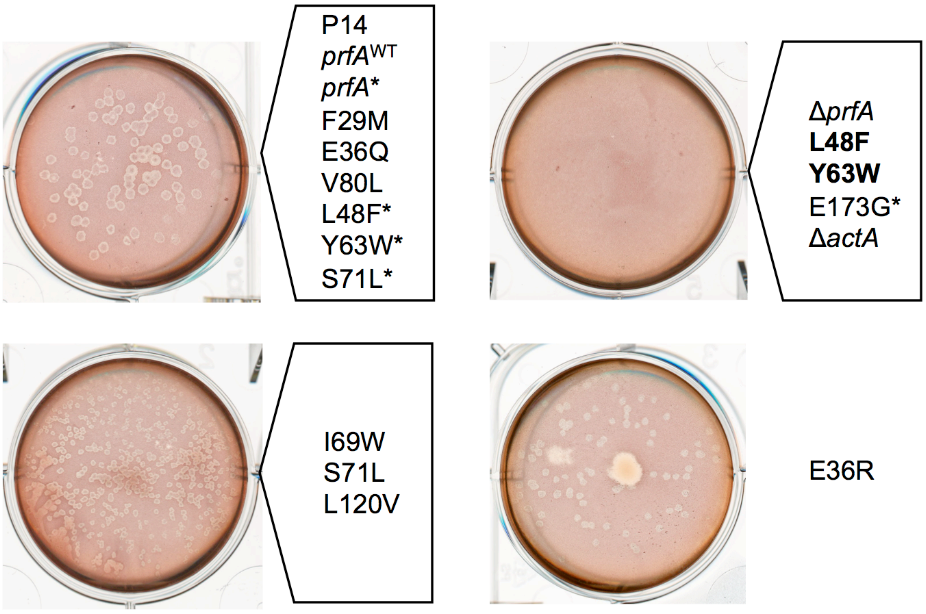

Fig. S8. Representative images illustrating the size of the plaques formed by *L. monocytogenes* expressing control and *prfA* mutant alleles in L929 mouse fibroblast monolayers 96 h after infection. The boxed bacteria show all a similar plaque phenotype (see Fig. 5). The *prfA*^{allo} L48F and Y63W bacteria did not form plaques, like the control Δ*prfA* strain (complemented with an empty vector) or the non-functional *prfA**E173G (*prfA**^{sup}) allele and the actin polymerisation-negative Δ*actA* mutant.

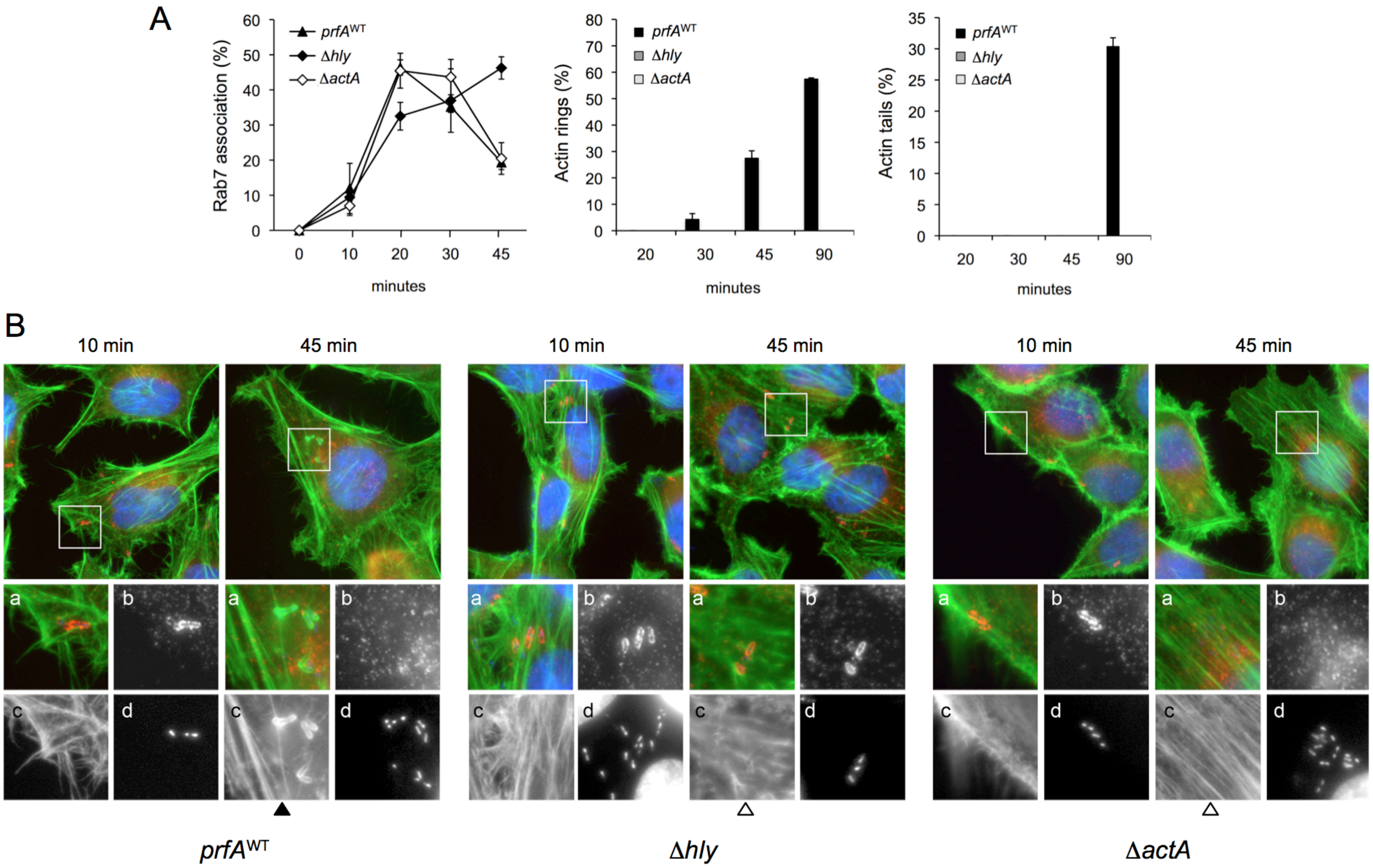

Fig. S9 (legend on next page).

Fig. S9. Vacuole escape assays with Δ*hly* and Δ*actA* controls in HeLa cells.

A. Vacuole escape dynamics determined by fluorescence microscopy as described in Fig. 7. Mean of three independent experiments ±SEM.

B. Representative fluorescence micrographs used to quantify vacuole escape in (A). White boxes in top panels (Rab7, F-actin and DAPI staining merge) indicate areas of interest and are shown as 2.5× magnified sections below: (a) image of boxed area; (b) Rab7 vacuole staining, which is clearly evident around bacteria 10 min after infection for *prfA*^{WT}, Δ*hly* and Δ*actA*, but only remains around the escape-defective Δ*hly* mutant after 45 min; (c) F-actin staining, which is not associated with any strain at 10 min, but present as actin rings at 45 min for *prfA*^{WT} (solid arrowhead) but not vacuole-bound Δ*hly* or the actin polymerisation-deficient Δ*actA* mutant (empty arrowheads); (d) DAPI staining, showing internalised bacteria. Images were originally captured at 630× magnification.

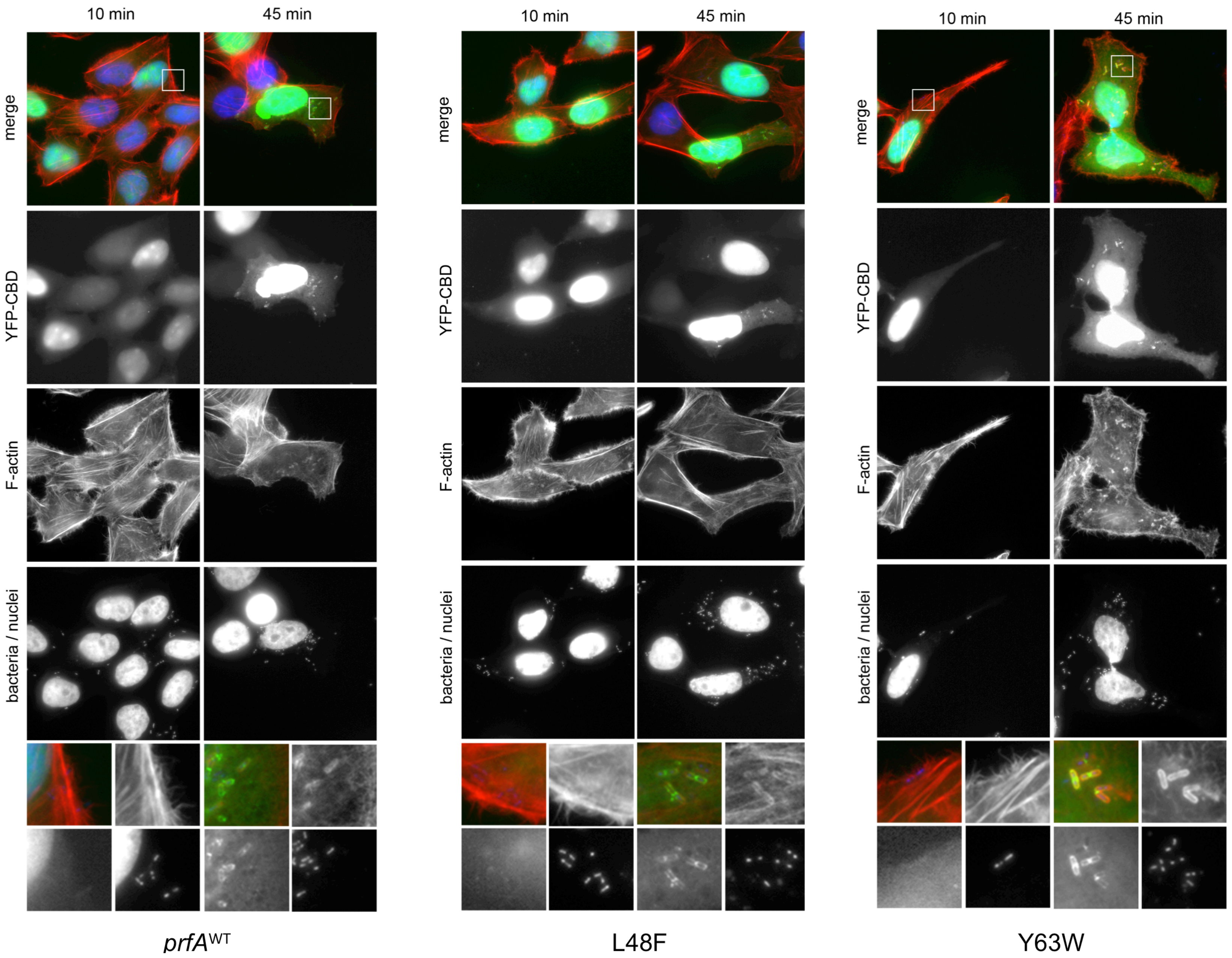

Fig. S10 (legend on next page).

Fig. S10. Vacuole escape assays of *prfA*^{allo} mutants using the YFP-CBD cytosolic probe. HeLa cells transfected with pEYFP-C1-CBD (Henry *et al*., 2006), encoding the cell wall-binding domain (CBD) from the *L. monocytogenes* phage A118 endolysin fused to yellow fluorescent protein (YFP), were infected with *prfA*^{WT} and *prfA*^{allo} L48F and Y63W strains and analysed by fluorescence microscopy at different time points. The panels are representative fluorescence micrographs of the CBD-YFP signal (green) plus AlexaFluor-546 phalloidin (red) and DAPI (blue) staining to visualise F-actin and internalised bacteria/cell nuclei, respectively. White boxes indicate areas of interest and are shown as 2.5× magnified sections beneath each panel for "merge" and individual channels. Accummulation of both the YFP-CBD cytosolic probe and F-actin can be observed around *prfA*^{WT}, L48F and Y63W bacteria after 45 min but not 10 min indicating phagosomal escape. Images were originally captured at 630× magnification.

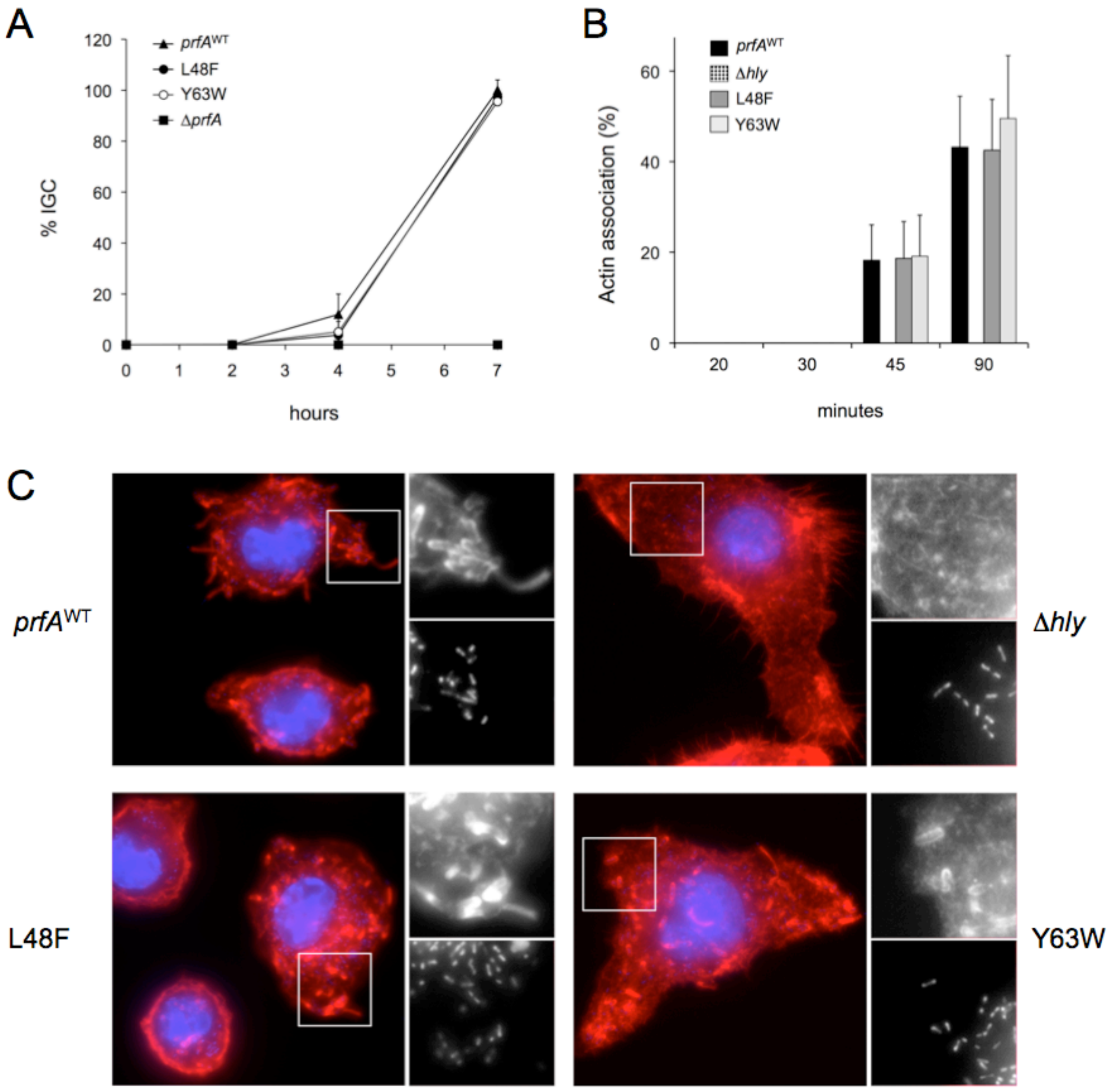

Fig. S11. Vacuole escape of *prfA*^{allo} L48F and Y63F mutants in J774 macrophages.
A. Intracellular growth. *L. monocytogenes* Δ*prfA* complemented with an empty vector or the *prfA*^{WT} allele were used as controls. Intracellular growth coefficient (IGC) expressed as percent of wild type at $t = 7$. Bacterial CFU counts per well at $t = 0$: *prfA*^{WT}, 1.7±0.3 × 10^5; *prfA*^{L48F}, 1.6±0.1 × 10^5; *prfA*^{Y63W}, 1.8±0.5 × 10^5; Δ*prfA,* 1.5±0.4 × 10^5. Mean of two duplicate experiments ±SEM.
B. Vacuole escape dynamics determined by quantification for bacterial association with F-actin, indicative of cytosolic localisation. See Fig. 7. Data for each time point are the mean percentage of five microscopic fields per experiment. Mean of three experiments ±SEM.
C. Representative fluorescence micrographs of vacuole escape at 90 minutes post infection. Main panels are an F-actin (red) and DAPI (blue) staining merge. White boxes indicate areas of interest and are shown as 2.5× magnified sections on the right: (top) F-actin staining, clearly visible in association with *prfA*^{WT} (rings and some tails) and *prfA*^{allo} strains (actin rings) but absent from the escape-deficient Δ*hly* mutant; (bottom) DAPI staining, showing internalised bacteria. Images were originally captured at 1000× magnification.

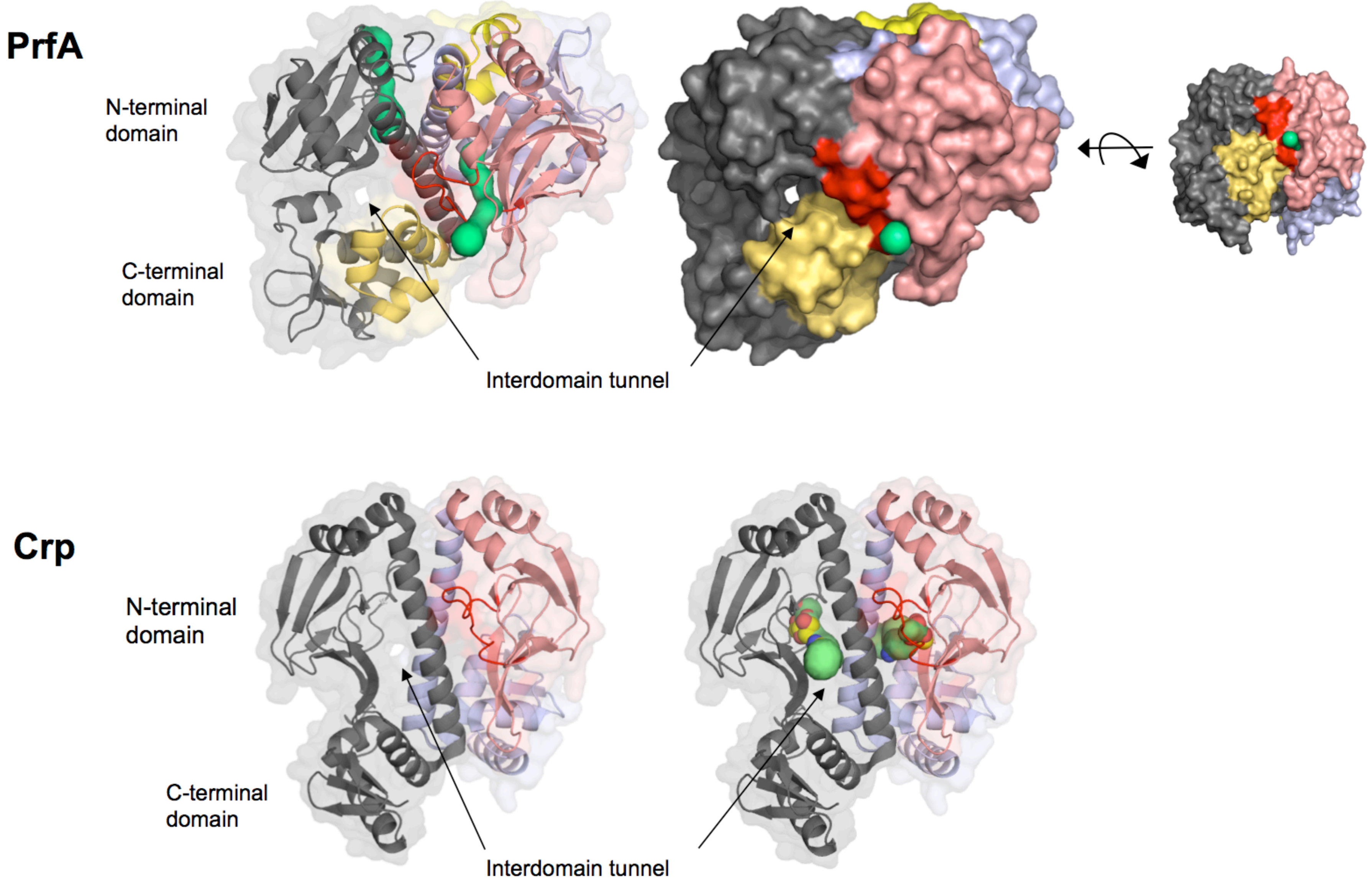

Fig. S12 (legend on next page).

Fig. S12. PrfA and cAMP-Crp complex dimer structures from Fig. 2 appropriately rotated to expose the interdomain tunnel. The N-terminal β-barrel pocket trajectories determined by CAVER (Petrek *et al.*, 2006) are represented in green. Note that whereas in Crp the entrance of the cAMP-binding pocket is through the interdomain tunnel, in PrfA the pocket mouth opens out at the opposite side of the β-barrel and does not coincide with the interdomain tunnel. This tunnel corresponds to the large hollow space left between the N- and C-terminal domains of $PrfA^{WT}$ (PDB code 2BEO) and which in surface representation (top right) appears as a large channel; it has two entries, traverses the molecule side to side and is not interconnected with the N-terminal domain pocket within the β-barrel. The interdomain tunnel is not visible in a surface representation of the $PrfA*^{G145S}$ 3D structure (PDB code 2BGC) (not shown), consistent with the observed "tightening" of the dimer around the C-helices associated with the G145S substitution (Table S1). This suggests that PrfA in "ON" state adopts a "closed" conformation similar to that reported for the cAMP-bound *E. coli* and *M. tuberculosis* Crp proteins (Popovych *et al.*, 2009; Reddy *et al.*, 2009).

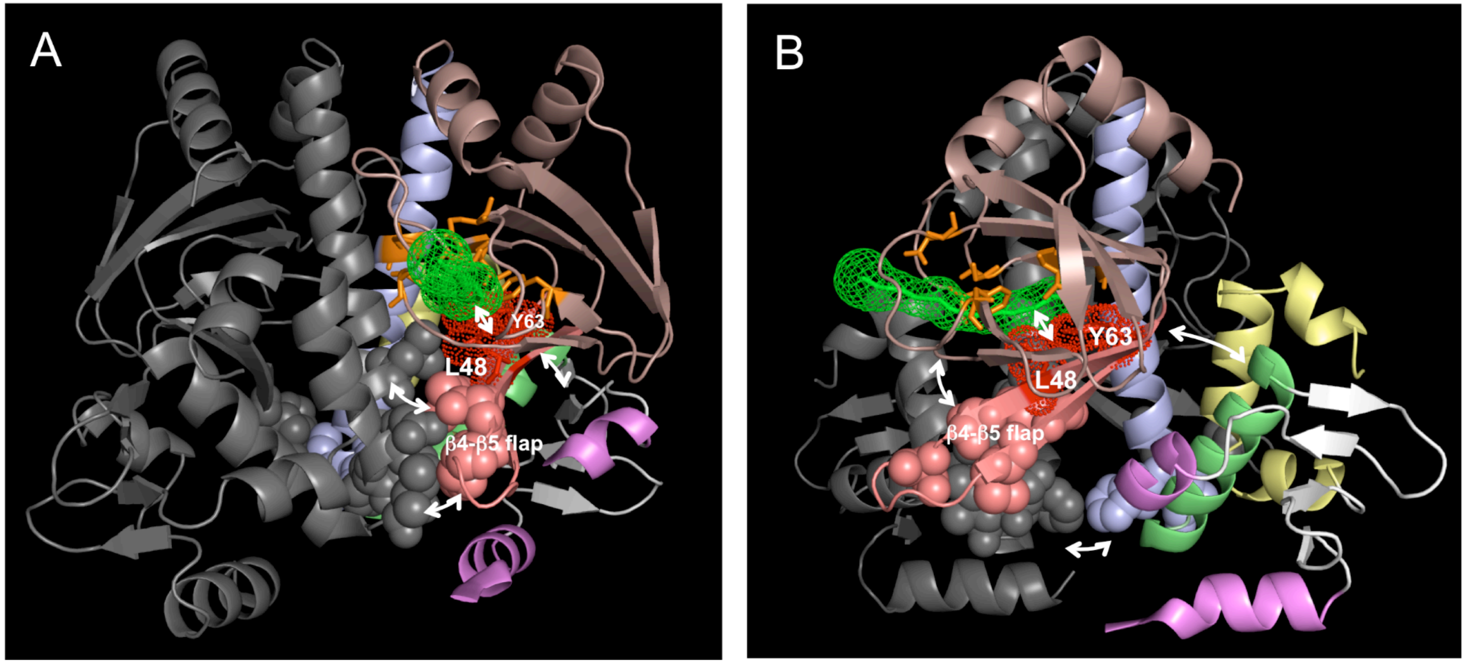

Fig. S13. Putative β-barrel "flap" contacts/motions potentially important for PrfA allosteric activation. Cartoon representation of the crystal structure of PrfA with the solvent-accessible pocket represented as a green mesh within the N-terminal β-barrel. One protomer is coloured by structural feature (β-barrel plus αA and αB in dark salmon, αC in light blue, αD helix [where the G145S PrfA* substitution is located] in green, HTH in magenta, C-terminal GHI α-helical bundle in yellow, rest of monomer in light grey), the opposite protomer is dark grey. The β4-β5 flap is highlighted in bright salmon. The pocket residues targeted by the activation-deficient $PrfA^{allo}$ substitutions, L48 on β4 strand and Y63 on β5 strand, are in red and their atomic volumes shown in dot representation. Their position in the structure suggests these residues are likely to play a critical role in β4-β5 flap motions upon binding of a ligand in the pocket. In spacefill representation: closely interacting residues from the flap and αC' from the opposite monomer, likely important in inter-protomer realignment upon ligand-triggered motion of the flap; see text; and F134 at the C-terminal end of αC and αC' (the phenyl rings of which are in van der Waals/hydrophobic contact at the monomer-monomer interface and may play a key role in the relative repositioning of the protomers upon the reported straightening/tightening of the C-helices associated with PrfA activation; Eiting *et al.*, 2005). White arrows indicate hypothetical motions/contacts potentially important in PrfA allosteric activation.

A. "Side" view of the PrfA dimer as in Fig. 2.

B. Same as in (A) with structure rotated leftwards by ≈90º on vertical axis.

Table S1. Accessible surface areas (ASA), gap volumes and gap volume indexes in the interfaces of the two monomers of $PrfA^{WT}$, $PrfA*^{G145S}$ and the pocket mutants most severely affected in intracellular activation (L48F, Y63W, S71L and L120V; Fig. 4). See Experimental Procedures and footnote [a] for more details.

**PrfA protein**	**ASA ($Å^2$)**	**Gap volume ($Å^3$)**	**Gap volume index**
$PrfA^{WT}$ [b]	2094	8839	2.11
$PrfA*^{G145S}$ [b]	2274	7546	1.66
$PrfA^{WT}$ model [c]	2070	9085	2.19
$PrfA*^{G145S}$ model [c]	2155	8670	2.01
$PrfA*^{G145S}$ model [d]	2170	7567	1.74
**L48F** [c,e]	2071	**9565**	**2.31**
Y63W [c]	2091	8468	2.02
S71L [c]	2115	8714	2.06
L120V [c]	2093	8981	2.15

[a] To assess the predictive accuracy of our structural modelling analysis, a theoretical model for $PrfA^{WT}$ was obtained based on the crystal structure of $PrfA^{WT}$; similarly, two models were obtained for $PrfA*^{G145S}$ using the crystal structures of $PrfA^{WT}$ and $PrfA*^{G145S}$. Both $PrfA*^{G145S}$ models predict a tightening of the dimer, as experimentally observed in the actual crystal structure, indicating that our models may give a reliable approximation to the structural features of the PrfA mutants.

[b] Actual values calculated from the crystal structures of $PrfA^{WT}$ (PDB code 2BEO) and $PrfA*^{G145S}$ (PDB code 2BGC). Note that in $PrfA*^{G145S}$ the ASA value increases whereas the gap volume decreases, indicating that the G145S substitution brings the two monomers closer to each other, "tightening up" the dimer.

[c] Modeled from $PrfA^{WT}$ crystal structure (PDB code 2BEO).

[d] Modeled from $PrfA*^{G145S}$ crystal structure (PDB code 2BGC).

[e] In L48F, the introduced phenylalanine is predicted to make close hydrophobic contacts with Y83 (2.9Å) and F29 (3.8Å) from the same monomer, and L128 (3.3Å) in the partner monomer, which represent steric clashes since the distances are smaller than the summed van der Waals radii (3.4 Å in the case of two carbon atoms). This implies that the accommodation of the L48F substitution involves adjustments in the PrfA structure and, likely also, in the relative position of the two protomers of the L48F dimer. The modelling data show that, while the accessible surface area (ASA) remains unchanged, the gap volume at the dimer interface is greater in $PrfA^{L48F}$ than in $PrfA^{WT}$, suggesting that the mutation moves the two monomers slightly apart.

Table S2. Bacterial strains and plasmids.

**Strain / plasmid** [a]	**Other designations** [b]	**Description**	**Source (reference)**	**Internal collection no.**
L. monocytogenes				
P14		Wild-type *L. monocytogenes* isolate (serovar 4b)	Our laboratory (Ripio *et al*., 1996)	PAM 14
Δ*prfA*		In-frame *prfA* deletion mutant of PAM 14	Our laboratory (Vega *et al*., 2004)	PAM 373
Δ*prfA* (pPL2)		Δ*prfA* complemented with pPL2 empty vector (complementation negative control)	This study	PAM 3293
Δ*prfA* (pPL2*prfA*^{WT})	*prfA*^{WT}	Δ*prfA* complemented with wild-type *prfA* allele	This study	PAM 3294
Δ*prfA* (pPL2*prfA**^{G145S})	*prfA**^{G145S}	Δ*prfA* complemented with mutant *prfA**^{G145S}	This study	PAM 3295
Δ*prfA* (pPL2*prfA*^{F29M})	*prfA*^{F29M}	Δ*prfA* complemented with mutant *prfA*^{F29M} allele	This study	PAM 3306
Δ*prfA* (pPL2*prfA*^{E36Q})	prfA^{E36Q}	Δ*prfA* complemented with mutant *prfA*^{E36Q} allele	This study	PAM 3304
Δ*prfA* (pPL2*prfA*^{E36R})	*prfA*^{E36R}	Δ*prfA* complemented with mutant *prfA*^{E36R} allele	This study	PAM 3305
Δ*prfA* (pPL2*prfA*^{L48F})	*prfA*^{L48F}	Δ*prfA* complemented with mutant *prfA*^{L48F} allele	This study	PAM 3299
Δ*prfA* (pPL2*prfA**^{L48F})	*prfA**$^{G145S/L48F}$	Δ*prfA* complemented with double mutant *prfA**$^{G145S/L48F}$ allele	This study	PAM 3300
Δ*prfA* (pPL2*prfA*^{Y63W})	*prfA*^{Y63W}	Δ*prfA* complemented with mutant *prfA*^{Y63W} allele	This study	PAM 3301
Δ*prfA* (pPL2*prfA**^{Y63W})	*prfA**$^{G145S/Y63W}$	Δ*prfA* complemented with double mutant *prfA**$^{G145S/Y63W}$ allele	This study	PAM 3302
Δ*prfA* (pPL2*prfA*^{I69W})	*prfA*^{I69W}	Δ*prfA* complemented with mutant *prfA*^{I69W} allele	This study	PAM 3303
Δ*prfA* (pPL2*prfA*^{S71L})	*prfA*^{S71L}	Δ*prfA* complemented with mutant *prfA*^{S71L} allele	This study	PAM 3297
Δ*prfA* (pPL2*prfA**^{S71L})	*prfA**$^{G145S/S71L}$	Δ*prfA* complemented with double mutant *prfA**$^{G145S/S71L}$ allele	This study	PAM 3298
Δ*prfA* (pPL2*prfA*^{V80L})	*prfA*^{V80L}	Δ*prfA* complemented with mutant *prfA*^{V80L} allele	This study	PAM 3296
Δ*prfA* (pPL2*prfA*^{L120V})	*prfA*^{L120V}	Δ*prfA* complemented with mutant *prfA*^{L120V} allele	This study	PAM 3307
Δ*prfA* (pPL2*prfA**^{A129T})		Δ*prfA* complemented with double mutant *prfA** $^{G145S/A129T}$ allele (dimerization-negative control PrfA mutant; see Table 1)	Our laboratory [c]	PAM 3318
Δ*prfA* (pPL2*prfA**^{E173G})	*prfA**^{sup}	Δ*prfA* complemented with double mutant *prfA**$^{G145S/E173G}$ allele (DNA-binding-negative control PrfA mutant)	Our laboratory [c]	PAM 3314
Δ*actA*		In-frame *actA* deletion mutant of *L. monocytogenes* P14 (cell-to-cell spread negative control)	Our laboratory	PAM 185
Δ*hly*		In-frame *hly* deletion mutant of *L. monocytogenes* P14 (vacuole escape negative control)	Our laboratory	PAM 3674

[a] Plasmid used for complementation is in brackets.

[b] Complemented strains were referred to in the text by the *prfA* allele they expressed.

[c] M. Bielecka *et al*., unpublished.

Table S2 (cont.). Bacterial strains and plasmids.

**Strain [a] / plasmid**	**Description / use**	**Source [reference]**	**Internal collection no.**
E. coli			
DH5α	Cloning host strain	Our laboratory	n.a.
BL21(DE_3)	Host strain for expression of recombinant proteins	Invitrogen	n.a.
BL21(DE_3) (pET28a)	Host strain with empty expression vector (negative control of recombinant PrfA production)	This study	PAM 3485
BL21(DE_3) (pET28a*prfA*^{WT})	Production of wild-type PrfA protein	This study	PAM 3486
BL21(DE_3) (pET28a*prfA**^{G145S})	Production of PrfA*^{G145S} mutant protein	This study	PAM 3487
BL21(DE_3) (pET28a*prfA*^{F29M})	Production of PrfA^{F29M} mutant protein	This study	PAM 3498
BL21(DE_3) (pET28a*prfA*^{E36Q})	Production of PrfA^{E36Q} mutant protein	This study	PAM 3496
BL21(DE_3) (pET28a*prfA*^{E36R})	Production of PrfA^{E36R} mutant protein	This study	PAM 3497
BL21(DE_3) (pET28a*prfA*^{L48F})	Production of PrfA^{L48F} mutant protein	This study	PAM 3491
BL21(DE_3) (pET28a*prfA**$^{G145S/L48F}$)	Production of PrfA*$^{G145S/L48F}$ double mutant protein	This study	PAM 3492
BL21(DE_3) (pET28a*prfA*^{Y63W})	Production of PrfA^{Y63W} mutant protein	This study	PAM 3493
BL21(DE_3) (pET28a*prfA*^{I69W})	Production of PrfA^{I69W} mutant protein	This study	PAM 3495
BL21(DE_3) (pET28a*prfA**$^{G145S/Y63W}$)	Production of PrfA*$^{G145S/Y63W}$ double mutant protein	This study	PAM 3494
BL21(DE_3) (pET28a*prfA*^{S71L})	Production of PrfA^{S71L} mutant protein	This study	PAM 3489
BL21(DE_3) (pET28a*prfA**$^{G145S/S71L}$)	Production of PrfA*$^{G145S/S71L}$ double mutant protein	This study	PAM 3490
BL21(DE_3) (pET28a*prfA*^{V80L})	Production of PrfA^{V80L} mutant protein	This study	PAM 3488
BL21(DE_3) (pET28a*prfA*^{L120V})	Production of PrfA^{L120V} mutant protein	This study	PAM 3499
BL21(DE_3) (pET28a*prfA**^{A129T})	Production of PrfA*$^{G145S/A129T}$ double mutant protein (dimerization-negative control PrfA)	Our laboratory [c]	PAM 3510
BL21(DE_3) (pET28a*prfA**$^{G145S/E173G}$)	Production of PrfA*$^{G145S/E173G}$ double mutant protein (DNA-binding-negative control PrfA)	Our laboratory [c]	PAM 3506
**Plasmid**			
pPL2	Integrative vector for stable single-copy gene complementation in *L. monocytogenes*.	M. Loessner (Lauer *et al*., 2002)	n.a.
pET28a	Expression vector for the production of N-terminal His-tagged recombinant proteins.	Novagen	n.a.
pEYFP-C1-CBD	Mammalian expression vector encoding the cytosolic marker YFP-CBD for vacuole escape assays	J. Swanson (Henry *et al*., 2006)	n.a.

[a] Plasmid used for complementation is in brackets.
[b] Some strains were referred to in the text by their relevant genotype / mutation.
[c] M. Bielecka *et al*., unpublished.

Table S3. Main oligonucleotides used in this study. Restriction site in italics, mutated codons in bold capitals, PrfA boxes in underlined capitals.

Primer	Sequence 5'→3' (comments)	Use
MR2*Kpn*I	ctag*GGTACC*caactaacatatattattcct (KpnI site)	PCR-amplification of *prfA* for insertion in pPL2 complementation vector.
MR10*Spe*I	ctag*ACTAGT*cttggtgaagcaatcgtacgc (SpeI site)	
prfAH1-P14	atga*CATATG*aacgctcaagcagaag (NdeI site)	PCR-amplification of *prfA* for insertion in pET28a expression vector.
prfAH2-P14	ga*GTCGAC*attgagacatcctgtttt (SalI site)	
F29Mforward	aacttatt**ATG**aaccaatggga	Mutagenesis Phe29Met of PrfA
F29Mreverse	tcccattggtt**CAT**aataagtt	
E36Qforward	atccacaa**CAA**tattgtatttt	Mutagenesis Gly36Gln of PrfA
E36Qreverse	aaaatacaata**TTG**ttgtggat	
E36Rforward	atccacaa**AGA**tattgtatttt	Mutagenesis Gly36Arg of PrfA
E36Rreverse	aaaatacaata**TCT**ttgtggat	
L48Fforward	tatcacaaag**TTT**acgagtatt	Mutagenesis Leu48Phe of PrfA
L48Freverse	aatactcgt**AAA**ctttgtgata	
Y63Wforward	ttacaatac**TGG**aaaggggctt	Mutagenesis Tyr63Trp of PrfA
Y63Wreverse	aagcccctttt**CCA**gtattgtaa	
I69Wforward	gctttcgtt**TGG**atgtctggct	Mutagenesis Ile69Trp of PrfA
I69Wreverse	agccagacat**CCA**aacgaaagc	
V80Lforward	agaaacatcg**TTA**ggctattat	Mutagenesis Val80Leu of PrfA
V80Lreverse	ataatagcc**TAA**cgatgtttct	
S71Lforward	cgttataatg**TTA**ggctttatt	Mutagenesis Ser71Leu of PrfA
S71Lreverse	aataaagcc**TAA**cattataacg	
L120Vforward	tccaaacc**GTT**caaaaacaagt	Mutagenesis Leu102Val of PrfA
L120Vreverse	acttgtttttg**AAC**ggtttgga	
E173Gforward	caatgcag**GGC**ttaggctattc	Mutagenesis Glu173Gly of PrfA
E173Greverse	gaatagcctaa**GCC**ctgcattg	
Promo40*plcA*-P14	Biotin-TEG-tgtccctttatcgtcgTTAACAAATGTTAAtgcctcgaca	Biacore DNA-binding affinity experiments (PrfA boxes of P*plcA*/*hly* and P*actA* promoters)
Promo40*actA*-P14	Biotin-TEG-ggtcggagttaactgaTTAACAAATGTTAGagaaaactta	
Promo40*plcA*REV	tgtcgaggcaTTAACATTTGTTAAcgacgataaagggaca	
Promo40*actA*REV	taagttttctCTAACATTTGTTAAtcagttaactccgacc	
*actA*38F	aagaaattgatcgcctagctgatt	*actA* gene expression analysis by RT-QPCR
*actA*109R	gtaaaaaacccgcatttcttgagt	
*actA*64P	FAM-tttcctgttcctctatctct-MGB-NFQ (Taqman probe)	
*plcA*11F	caggtacacatgatacgatgagctataa	*plcA* gene expression analysis by RT-QPCR
*plcA*99R	gtacaatgacatcgtttgtgtttgag	
*plcA*P	FAM-agtggtttggttaatgtcc-MGB-NFQ (Taqman probe)	
T3	attaaccctcactaaaggga	Competitive virulence assay
prfA3F	ccctacaaaaacaagtttcata	
prfA2R	attcttgctcagtagttctttta	
